# VDAC3 as a sensor of oxidative state of the intermembrane space of mitochondria: the putative role of cysteine residue modifications

**DOI:** 10.18632/oncotarget.6850

**Published:** 2016-01-08

**Authors:** Simona Reina, Vanessa Checchetto, Rosaria Saletti, Ankit Gupta, Deepti Chaturvedi, Carlo Guardiani, Francesca Guarino, Mariano Andrea Scorciapino, Andrea Magrì, Salvatore Foti, Matteo Ceccarelli, Angela Anna Messina, Radhakrishnan Mahalakshmi, Ildiko Szabo, Vito De Pinto

**Affiliations:** ^1^ Department of Biomedicine and Biotechnology BIOMETEC, Section of Biology and Genetics, University of Catania, Catania, Italy; ^2^ National Institute for Biomembranes and Biosystems, Section of Catania, Catania, Italy; ^3^ Department of Biology, University of Padova, Padova, Italy; ^4^ CNR Institute of Neurosciences, Padova, Italy; ^5^ Department of Biomedical Sciences, University of Padova, Padova, Italy; ^6^ Department of Chemical Sciences, Mass Spectrometry Unit, University of Catania, Catania, Italy; ^7^ Molecular Biophysics Laboratory, Department of Biological Sciences, Indian Institute of Science Education and Research, Bhopal, India; ^8^ Department of Physics, University of Cagliari, Cagliari, Italy; ^9^ Department of Biomedical Sciences, Biochemistry Unit, University of Cagliari, Cagliari, Italy; ^10^ Istituto Officina dei Materiali del Consiglio Nazionale delle Ricerche (IOM-CNR), UOS, Trieste, Italy; ^11^ Department of Biological, Geological and Environmental Sciences, Section of Molecular Biology, University of Catania, Catania, Italy; ^12^ National Institute for Biomembranes and Biosystems, Section of Catania, Catania, Italy

**Keywords:** VDACs, cysteine oxidation, disulfide bridge, mitochondrial intermembrane space, mass spectrometry

## Abstract

Voltage-Dependent Anion selective Channels (VDAC) are pore-forming mitochondrial outer membrane proteins. In mammals VDAC3, the least characterized isoform, presents a set of cysteines predicted to be exposed toward the intermembrane space. We find that cysteines in VDAC3 can stay in different oxidation states. This was preliminary observed when, in our experimental conditions, completely lacking any reducing agent, VDAC3 presented a pattern of slightly different electrophoretic mobilities. This observation holds true both for rat liver mitochondrial VDAC3 and for recombinant and refolded human VDAC3. Mass spectroscopy revealed that cysteines 2 and 8 can form a disulfide bridge in native VDAC3. Single or combined site-directed mutagenesis of cysteines 2, 8 and 122 showed that the protein mobility in SDS-PAGE is influenced by the presence of cysteine and by the redox status. In addition, cysteines 2, 8 and 122 are involved in the stability control of the pore as shown by electrophysiology, complementation assays and chemico-physical characterization. Furthermore, a positive correlation between the pore conductance of the mutants and their ability to complement the growth of porin-less yeast mutant cells was found. Our work provides evidence for a complex oxidation pattern of a mitochondrial protein not directly involved in electron transport. The most likely biological meaning of this behavior is to buffer the ROS load and keep track of the redox level in the inter-membrane space, eventually signaling it through conformational changes.

## INTRODUCTION

The voltage-dependent anion selective channels (VDAC) are a family of proteins whose primary role is to transport hydrophilic metabolites and molecules through the mitochondrial outer membrane (MOM). In mammals evolution yielded three distinct VDAC isoforms, but the specific biological function for the presence of three distinct genes is not clear yet [1]. Among them, VDAC1 is the best characterized isoform [2, 3, 4] which was shown to play an important role in several cell processes, including apoptosis [5, 6], calcium homeostasis [7], in diseases such as cancer [8] and in general it takes part in controlling the energetic metabolism [9]. Deficiency of VDAC1 has been associated with a lethal encephalomyopathy [10]. VDACs contain a common amphipathic trans-membrane β-barrel motif formed by 19 β-strands and a short N-terminal sequence that has a partial character of α-helix. The N-terminal sequence exhibits differences between the three isoforms. In the available 3D structures of VDAC1 [11, 12, 13] and of VDAC2 [14], the N-terminal domain presents an α-helical folding among residues 6-20 and runs along the channel making contacts with the internal face of the pore. It has been proposed that the channel can be regulated by a gating mechanism involving the movement of the N-terminal domain [15]. VDAC3, although shares about 60-70% sequence identity with VDAC1 and VDAC2, remains the least known isoform. Contrary to VDAC1 and VDAC2, VDAC3 is indeed unable to recover the growth defect of a yeast strain devoid of the endogenous VDAC (*Δpor1*) on non fermentable carbon sources at 37°C [16, 17]. VDAC isoform 3 was even considered unable to form channels in artificial membranes [18]. Recently, an electrophysiological activity, characterized by very low conductance levels, was described for recombinant human VDAC3 (hVDAC3) [19]. In a previous work [20], we demonstrated that the replacement of the first 20 amino acids of the N-terminal region of hVDAC3 with the corresponding residues of hVDAC1 was sufficient to radically change the features of the protein. The swapping chimera hN1-VDAC3 restored the growth of *Δpor1* yeast cell on glycerol at 37°C [20] and, upon reconstitution in planar bilayers, inserted easily into the membrane, showing a channel conductance comparable to VDAC1 [21]. This result prompted us to analyze in detail the N-terminal sequence of VDAC3. Human VDAC3 has six cysteines (seven in rat), two of them in the N-terminal domain, while VDAC1 only has two (no one of them in the N-terminal). VDAC2 too has a large number of cysteines, nine in human and mouse, but only one of them is conserved in the N-terminal in position corresponding to VDAC3. Based on the recent determination of the transmembrane topology of VDAC [22], four of the six cysteines of hVDAC3 are predicted to be located in the connection loops between β-strands, protruding towards the intermembrane space (IMS) (Cys36, Cys65, Cys122, Cys229) and two are located in the N-terminal domain (Cys 2 and Cys8), with the more proximal of them also exposed to the IMS (Cys2). When a cysteine residue is exposed on the surface of a protein, it can interact with H-bond partners (e.g. water molecules). These interactions polarize the bond and induce a significant decrease of pKa. Exposed cysteines can thus easily shift from the reduced to the oxidized form in response to small fluctuations of the pH. In the IMS reactive oxygen species (ROS) are poured by the complex III [23] and by monoamine oxidase [24] and the pH is more acidic because of the pumping activity of the electron chain complexes [25]. It is therefore remarkable that in hVDAC3 most of the cysteine residues are exposed to the oxidative and slightly acidic environment of the IMS. The exposure of the hVDAC3 cysteines suggests that they should be highly reactive and available to bear modifications of their oxidation state. In this work, we have investigated the redox state of cysteines both in a recombinant human VDAC3 protein and in rat mitochondrial VDAC3. We studied the results of cysteine removal in terms of the modification of the biological and biophysical features of the protein.

## RESULTS

### Reduction of VDAC3 cysteines modifies the protein electrophoretic pattern

**Figure 1:**
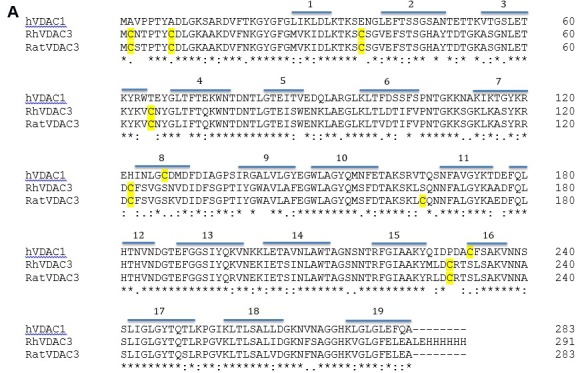

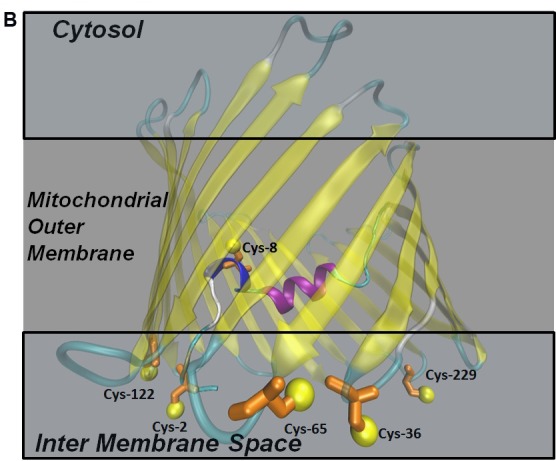

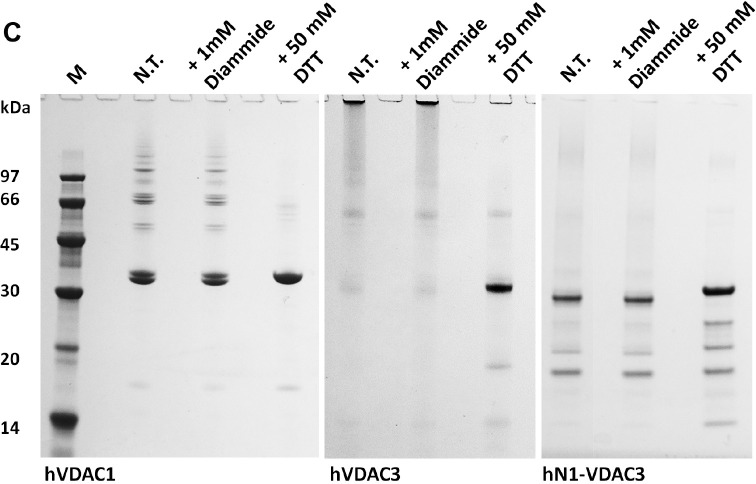
Cysteine content influences VDAC3 features **A.** VDAC3 has more cysteine residues than VDAC1. Recombinant human VDAC3 (RhVDAC3), rat VDAC3 (ratVDAC3), human VDAC1 (hVDAC1) were aligned with Clustal omega 1.2.1. In yellow are highlighted the cysteine residues. The numbered bars indicate predicted β-strands. RhVDAC3 (recombinant human VDAC3) shows also the poly-His tag. Notice that the cysteine residues in VDAC3 never are in β-strands, at a variance from VDAC1. **B.** A structural model of human VDAC3 showing the localization of cysteine residues, represented in orange-licorice and with the sulfur atoms as yellow balls. The homology model of hVDAC3 was obtained from the template X-ray structure of mouseVDAC1 (PDB:3EMN). The model was built using MODELLER 9v5 and the image by means of the program VMD 1.9.2. Assuming the sidedness reported in [22], cysteine residues in VDAC3 protrude toward the intermembrane space. **C.** Cysteine content changes protein electrophoretic mobility. hVDAC1, hVDAC3 and hN1-VDAC3 were pre-incubated with 1 mM diamide or 50 mM DTT and loaded on a 12% Nu-Page Novex gel without any reducing agent. A not-treated sample was used as control. 1X MES buffer was used for the run. hVDAC1 shows the expected electrophoretic migration. hVDAC3 is almost completely aggregated unless it is reduced with DTT. hN1-VDAC3 behaves similarly to hVDAC1 and does not aggregate at all.

A comparison between VDAC3 and VDAC1 highlights the presence, in VDAC3, of a higher number of cysteines (Figure 1A). In Figure 1B a structural model of hVDAC3 (obtained from [26]) shows the localization of cysteines in the predicted structure. As mentioned above, four out of six cysteine residues (Cys36, Cys65, Cys122, Cys229) are located in loops connecting *β*-strands and are thus exposed to the intermembrane space (IMS). Moreover, even if the N-terminal tail is localized inside the pore, as found in the template structure [12], in the homology model Cys2 is oriented toward the intermembrane space [26]. The exposure of the hVDAC3 cysteines suggests that they are highly accessible to soluble oxidative molecules and for this reason they are possibly linked to some specific biological function.

For a preliminary investigation of the redox state of VDAC3 cysteines, recombinant hVDAC1, hVDAC3 and the chimera hN1-VDAC3 [20] were expressed, refolded and probed to be active in reconstitution experiments (see below), and then subjected *in vitro* to reducing or oxidizing conditions (Figure 1C). To avoid any potential interference in the light of our purposes, DTT or any reducing chemical was omitted throughout the whole procedure unless clearly described. For *in vitro* modifications, aliquots of the recombinant proteins were either incubated with the oxidizing agent diamide, or with the reducing DTT at 4°C, acetone-precipitated and next ran in SDS-PAGE without reducing agents. While hVDAC1 showed the expected electrophoretic migration corresponding to the estimated Mr (35 kDa), in the various conditions, hVDAC3 was almost completely present in the top of the gel, most probably in an aggregated form, even though minor bands were visible at the expected Mr of the monomeric form and at intermediate migration levels. Oxidation by diamide did not relieve the aggregation, while reduction by DTT restored hVDAC3 migration at the expected molecular weight of 35 kDa, as a single homogeneous band (Figure 1C). The presence of double bands does not indicate truncated forms of the protein according to Mass Spectrometry (see below) and to the effect of reduction by DTT. VDAC3 has been shown to migrate faster than VDAC1 in reducing SDS-PAGE [27]. Instead, the presence of a faint band above the major one suggests that even the recombinant protein carries slight oxidation, possibly responsible for the band duplication. Other proteins have been shown to modify their electrophoretic mobility upon cysteine oxidation [28]. hN1-VDAC3, lacking the two cysteines in the N-terminus, in reducing conditions had the same mobility of VDAC1, and completely entered into the gel without the addition of DTT (Figure 1C). This result indicates that oxidized VDAC3 tends to oxidize easily and aggregate; the aggregation can be dissolved by a reducing agent. In this artificial system the main responsible for the aggregation of recombinant hVDAC3 appear to be C2 and C8. Similar results were obtained when the VDAC3 was in 8M urea, before the refolding procedure (not shown).

### The removal of specific cysteines changes electrophoretic migration of hVDAC3

**Figure 2:**
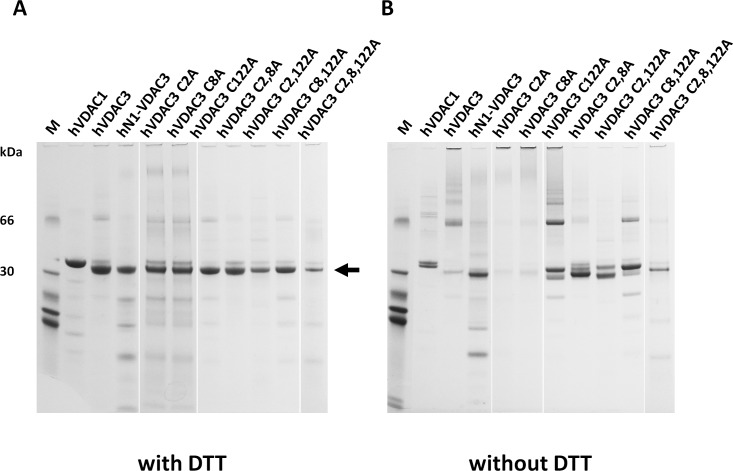
Selective cysteine mutations change the electrophoretic behavior and the ability to complement a porin-defective yeast strain **A.** Reduced hVDAC3 mutants show the same electrophoretic mobility. Recombinant refolded hVDAC3 Cys-- > Ala mutants were reduced with 50 mM DTT and run on a 12% NuPAGE Novex. M: Molecular weight markers: the migration of 66 and 30 kDa marker proteins is shown. The arrow indicates the VDAC3 mobility. **B.** Not reduced hVDAC3 mutants show different electrophoretic mobilities. The protein samples showed in A were run in a 12% Nu-Page Novex gel in non-reducing conditions and without any prior reducing incubation.

Suspecting a key role for the N-terminal cysteines in the electrophoretic mobility of hVDAC3, site-directed mutagenesis was used to substitute these amino acids with alanine residues. In particular we focused on cysteine 2, 8 and 122, the latter two being the closest residues to C2 on the basis of our predictions (Figure 1B). Single, double and triple mutant hVDAC3 sequences were cloned in pET21a vector, for protein expression in *E. coli*. Following purification, recombinant hVDAC3 mutants were refolded using an established protocol demonstrated to form active pores, and run in SDS-PAGE in the presence or absence of reducing agents (Figure 2A-2B). Any mutant protein, reduced with DTT, had the same electrophoretic mobility (Figure 2A). Conversely, without the reducing agent, each protein showed peculiar migration(s) in the gel (Figure 2B). The two VDAC3 single mutants C2A and C8A showed the same electrophoretic pattern of hVDAC3 wild type, indicating that the ablation of each of these cysteines, alone, does not change the proteins' electrophoretic mobility. In the same conditions, the single mutation C122A showed a complex pattern with several bands at various apparent Mr and, among them, a band clearly corresponding to a possible dimer. The deletion of two (C2,8A, C2,122A, C8,122A) or three cysteines (C2,8,122A) essentially reestablished a more homogenous electrophoretic migration, decreasing the protein aggregation. These data were confirmed in other gels where each mutant protein was overloaded and subjected to an oxidizing treatment with diamide (Suppl. Figure 1).

### The cysteine residues affect hVDAC3 activity in *Δpor1* yeast cells

**Figure 3:**
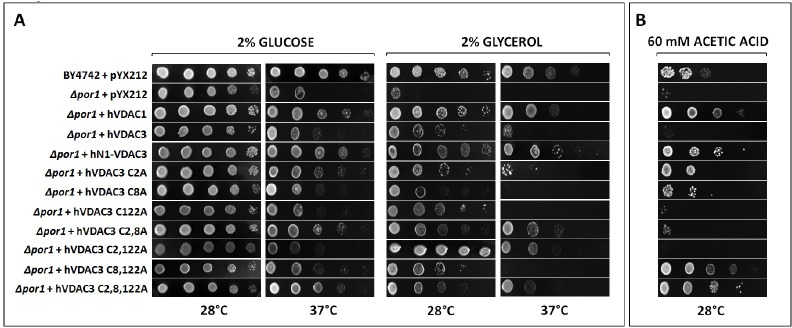
**A.** hVDAC3 cysteine mutants have different behaviors in Δpor1 yeast complementation assay. *Δpor1* yeast transformed with hVDAC3 containing different set of Cys-to-Ala mutations were tested in a drop-serial dilution assay. hVDAC1, hN1-VDAC3 and hVDAC3 were used as controls. Cells were plated on media containing glucose or glycerol as the sole carbon source and incubated at 28°C and 37°C for 4-5 days. Only C2,8A, C2,122A and C2,8,122A mutants enable *Δpor1* to grow on glycerol at 37°C. A partial recovery is also observed with C2A mutant. **B.** hVDAC3 mutants react differently to the pro-apoptotic stimulus of acetic acid. In the presence of 60 mM acetic acid, only hVDAC3 C2A, C8A, C8,122A, and C2,8,122A allowed the yeast growth. The figure shows a representative panel of experiments.

As already mentioned, unlike VDAC1 and VDAC2, VDAC3 is not able to allow *Δpor1* yeast cells to grow on non fermentable carbon sources like glycerol at 37°C [16]. Therefore, to evaluate the effects of the mutagenesis of cysteine residues on protein activity *in vivo*, hVDAC3 cysteine mutants were cloned in pYX212 vector for protein expression in *S. cerevisiae*, and used in complementation assays together with hVDAC1, hVDAC3 and hN1-VDAC3 as controls (Figure 3A). While no particular difference was observed on glucose at 28°C, most of hVDAC3 mutants improved *Δpor1* cell growth on glucose at 37°C and glycerol at 28°C compared to the control. Conversely, only *Δpor1* cells transformed with hVDAC3 double mutants C2,8A and C2,122A and the triple mutant C2,8,122A were able to grow on glycerol at 37°C, the most restrictive condition. A partial recovery was also observed with C2A. Interestingly, hVDAC3 C8,122A did not show the same behavior as the other two double mutants, suggesting a particular relevance of C2 in channel activity. Its removal indeed, together with C8 and/or C122, not only allowed hVDAC3 to complement *Δpor1* growth defect, but also strongly lowered the amount of aggregated protein in non-reducing conditions (Suppl. Figure 1).

The effect of pro-apoptotic agents on *Δpor1* cells transformed with the various mutants was also investigated. As already reported [29], acetic acid triggers apoptosis in yeast through mitochondrial ROS accumulation and decrease in mitochondrial respiration. The lack of endogenous VDAC1 in yeast makes the cells much more sensitive to acetic acid than the wild type ones [30]. Whereas the transformation with hVDAC1 and hN1-VDAC3 increased the resistance of *Δpor1* strain to 60 mM acetic acid, hVDAC3 failed to do the same. The analysis of hVDAC3 mutants growth on acetic acid is shown in Figure 3B. It is interesting to note that the removal of a single cysteine of the N-terminal domain (hVDAC3 mutants C2A and C8A) allows *Δpor1* yeast cell to grow on medium containing acetic acid, while the simultaneous removal of both residues does not determine the same effect. This is in contrast with the results obtained for the chimera hN1-VDAC3, which increases the resistance of *Δpor1* strain to acetic acid although without cysteine 2 and 8. One possible explanation is that the amino acids around the hVDAC1 N-terminus, being different from that of hVDAC3, could influence the effect of oxidative stress on these residues. The absence of the cysteine 122 alone or together with the cysteine 2 is not sufficient to change the behavior of hVDAC3 towards acetic acid. On the contrary, the removal of the cysteine 122 together with the residue 8 or the simultaneous absence of all three cysteine residues (hVDAC3 mutant C2,8,122A) allows *Δpor1* yeast cells to grow on 60 mM acetic acid. These data highlight that the different cysteines probably play distinct roles in the response to mitochondrial ROS.

### The cysteine residues oxidation influences the electrophysiological features of VDAC3

The human VDAC3 Cys mutants expressed *in vitro* were refolded and studied in a planar bilayer set-up to detect their ability to form conductive pores in the artificial membrane. We previously reported a characterization of recombinant hVDAC3 where we detected ion channels with a substantially lower conductance than VDAC1 [19]. Data reported in the present paper (Figures 1C and 2A) suggest that the preparation we used for our study with recombinant hVDAC3 is actually a mixture of proteins each displaying a different cysteine redox state. The lack of redox homogeneity in the protein sample might be the cause of the poor incorporation of the protein in artificial membrane or of the various pore-conductance states [18-19].

We thus performed a careful analysis of the action of reducing agents on the pore-forming activity of VDAC3. The recombinant hVDAC3 we used was isolated under non-reducing conditions. However, since the -SH groups of the cysteine might be oxidized at various extent as shown in this work and this may have an impact on the single channel conductance, we performed bilayer experiments with VDAC3 pre-incubated with reducing agents (DTT and β-mercaptoethanol) or treated with these agents during the experiment at pH 7.0 (*n* = 8) (Suppl. Figure 2A-2B). In 41 independent experiments carried out at pH 7.0 with VDAC3 in 1 M KCl without addition of reducing agents, we observed activity with well-resolved openings and closures (gating) and with a single channel conductance ranging between 90 and 140 pS, in accordance with our previous results [19]. In rare cases, conductance values larger than the unitary one were observed, and, most probably, this was due to the cooperative gating illustrated in Suppl. Figure 2D (see also respective histogram where numerous distinct current levels co-exist throughout the experiment). Finally, in 4 experiments out of these 41, single 3 nS steps have been observed more than half an hour after the incorporation of the small-conductance form, and only if the membrane was kept at ±10 mV potential for the whole experiment. However, when already the purification and refolding has been carried out in the presence of DTT, the situation was different: in three bilayer experiments where DTT was included also in the experimental medium, we detected events with high conductance levels typical of VDAC1 (Suppl. Figure 2E) (for reviews see [31, 32]), also in accordance with the findings of Okazaki [33]. Please note however, that the redox state of the IMS renders unlikely operation of VDAC3 in a fully reduced state *in vivo*.

**Figure 4:**
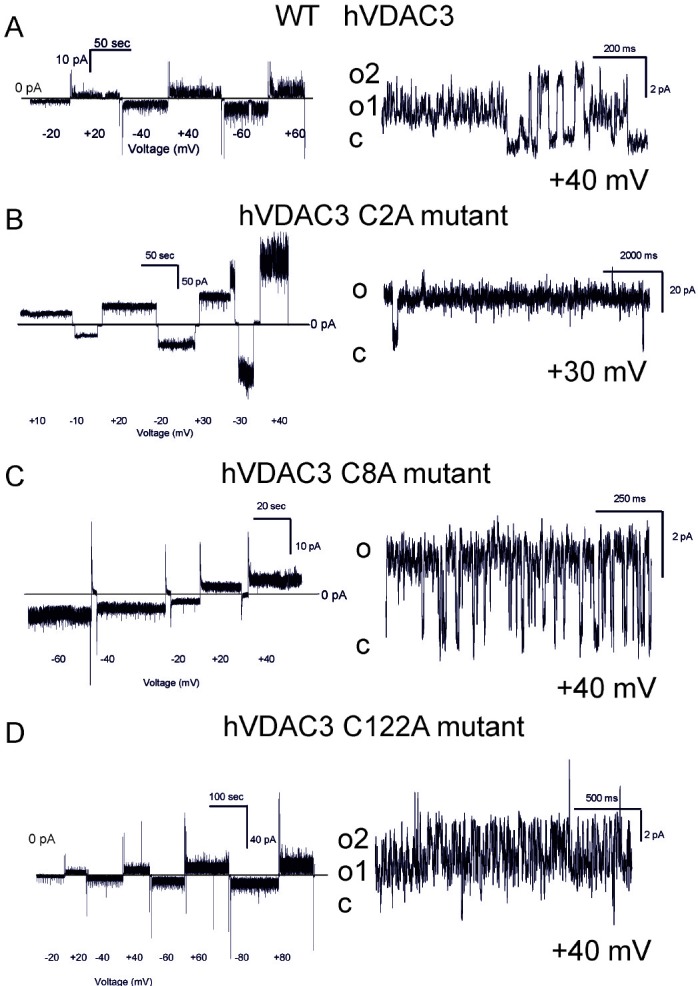

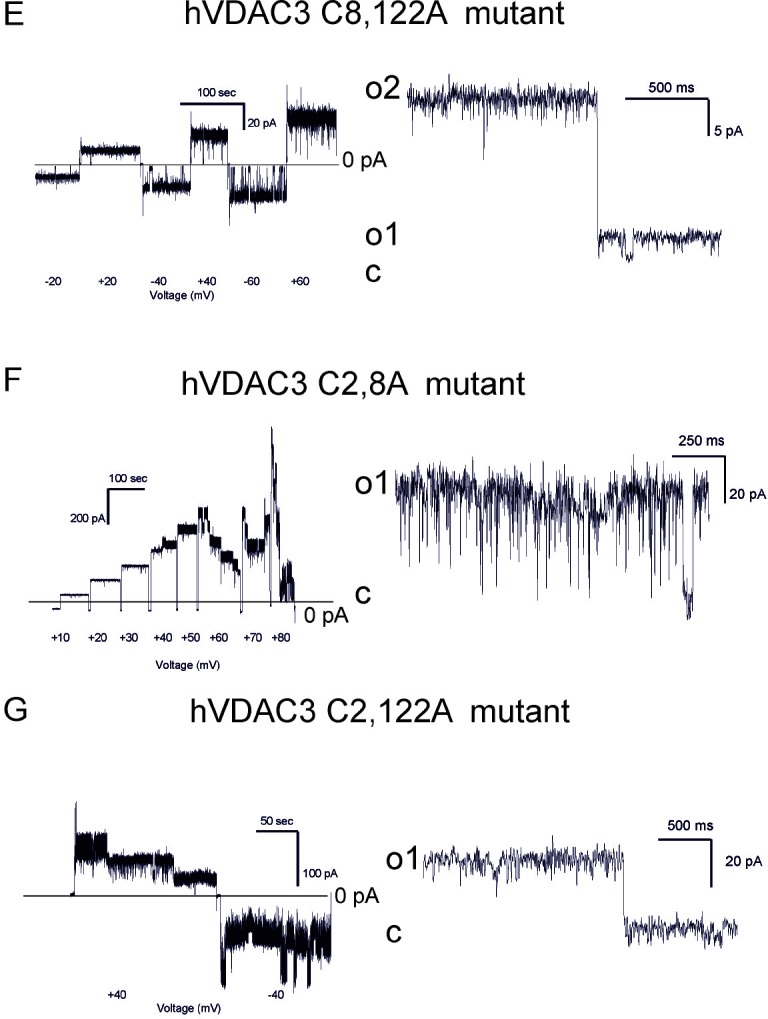
Conductance and voltage-dependence of hVDAC3 single, double and triple Cys>Ala mutants **A.**-**D**. Representative current traces recorded for (A): WT hVDAC3; (B): C2A; (C): C8A; (D): C122A. The mutants activities were recorded at varying applied potentials (left panels) and in an expanded time scale at the indicated voltages at pH 7.0 in 1 M KCl (right panels). The voltage protocol applied (left panels) allowed us to record channel activity at steady-state voltage and to better visualize the kinetic behavior of the channel (right panels). Tendency to close at higher voltages than ±30 mV was never observed, indicating that the voltage-dependence of wt VDAC3 was maintained ((A) and see also [19]). Open and closed states are indicated with “o” and “c”, respectively. In (A) conductance is 90 pS. In (B) activity recorded for C2A reveals a conductance of 800 pS and long-lasting open states (o). In (C) a conductance of 100 pS was observed for C8A, while in (D) activity recorded at Vcis of +40 mV with a conductance of 90 pS can be observed for C122A. **E.**-**G**. Traces for the indicated double mutants reported as in (A-D). The observed conductances for the examples shown are as follows: (E): C8,122A: 400 pS (for open level o2, Vcis +40 mV); (F): C2,8A: 1,2 nS (at Vcis +40 mV); (G): C2,122A: 800 pS (Vcis +40 mV). In (E) also a smaller conductance level (o1) can be observed. Both C2,8A and C2,122A mutants tend to close at potentials higher than ±30 mV, suggesting that modification of cysteine residue 2 may be an important element of VDAC3 channel activity modulation. Similar activities have been observed in at least 4 different experiments for each mutant. **H.** Upper current traces showing representative activity from an experiment with the triple mutant hVDAC3 C2,8,122A recorded in 1 M KCl at pH 7.0 in non-reducing conditions at the indicated voltages. **I.** As in H from the same experiment. A clear tendency to close at voltages higher than ±60 mV has been observed in other 5 experiments. In the presented experiment, the channel was operating in the low-conductance state at lower potentials, while at + 40 mV simultaneous opening of more channels was observable. **J.** At −60 mV and at + 60 mV an overall conductance of 4.6 nS was measured, as shown also in the amplitude histogram, but single channel activities with 1 nS conductance are well resolvable both in the current trace and in the histogram. **K.** A representative current trace from another experiment with a conductance of 1 nS. Similar data were obtained in 6 experiments.

Next, the hVDAC3 cysteine mutants' pore-forming activity was characterized at the bilayer set-up (number of experiments ranging from 4 to 16), revealing that at pH 7.0 (without addition of reducing agents), different Cys residues have distinct roles in the stabilization of the high-conducting state of VDAC3. Table 1 summarizes the conductance values recorded with the various mutants as well as their ability to complement the growth of the yeast mutants. Figure 4 shows the representative single channel behavior of wtVDAC3 and of individual Cys- > Ala mutants. C2A mutation increased the single channel conductance, with values ranging from 750 to 900 pS in the various experiments, as determined from the respective amplitude histograms (not shown) (*n* = 4 independent experiments) (Figure 4B). Furthermore, the channel was operating mostly in the open state and displayed brief closures. C8A mutation, instead, reproducibly resulted in a small-conductance activity with g values ranging from 70 to 100 pS (*n* = 4 independent experiments) (Figure 4C) i.e. similar to those of wt hVDAC3. In addition, channel activity was characterized by fast closures and openings (flickering), indicating that this mutation affects the kinetic behavior of VDAC3. C122A mutant, similarly to C8A, did not induced conductance or voltage-dependence or kinetic behavior changes with respect to VDAC3 [19]. Indeed, the channel was active also at high potentials (Figure 4D). In the double mutant C8,122A, VDAC3 tended to adopt a higher conductance state (ranging between 350 and 400 pS, *n* = 8) and slow kinetics (Figure 4E), suggesting that these two residues might contribute to keep the pore in a relatively low conductance state. Please note that the individual mutation of C8 and C122 did not strongly alter the characteristics of the wt channel instead. The C2,8A double mutant had a significantly higher single channel conductance than wt, with an average value of 1.3-1.4 nS (*n* = 16). Figure 4F illustrates the typical behavior of this double mutant: with an applied voltage up to +/−60 mV the channel operated in a completely open state. The simultaneous opening of several channels in the same membrane yielded whole-membrane conductance levels ranging up to 10 nS.

At higher potentials, where the channels start to close, it is well visible that closures took place in distinct steps of events with a conductance of 1.3 nS. The C2,122A mutant also displayed a high conductance (between 850 and 900 pS in the various experiments, *n* = 4) and fast gating, in a reproducible way (Figure 4G). Thus, the experiments with double mutants indicated that these three cysteine residues are subject to oxidation(s) affecting the channel behavior. Finally, we investigated the properties of the triple C2,8,122A mutant (Figure 4H-4K). In the case of the triple mutant, we repeatedly observed that the pore adopted lower conductance states (around 200 pS) mainly at low membrane potentials, but also a higher conductance state with a g value around 1 nS (Figure 4I). The pore again tends to close at potentials higher than ±80 mV. Thus, either single or multiple mutations of the VDAC3 selected cysteine recapitulate the VDAC1 typical high-conductance state, although the voltage-dependence remains different (VDAC1 starts to show closing events at applied voltages of 30-40 mV).

**Table 1:** Correlation between conductance values and complementation in yeast

Protein	Yeast complementation	Most frequently observed conductance states	Voltage dependence
hVDAC1	YES	3000 pS (n=6)	Voltage-dependet, tendency to close at applied voltages >30 mV
hVDAC3	NO	90 pS (n=4)	No Voltage-dependence, no closure even at high applied potentials
N1-hVDAC3	YES	1000 pS (n=12)	Tendency to close only at high applied voltages
hVDAC3 C2A	PARTIAL	750-900 pS (*n* = 4)	Tendency to close only at high applied voltages (V>60 mV)
hVDAC3 C8A	NO	70-100 pS (*n* = 4)	No Voltage-dependence
hVDAC3 C122A	NO	150 pS (*n* = 6)	No Voltage-dependence
hVDAC3 C2-8A	YES	1200-1400 pS (*n* = 16)	Tendency to close only at high applied voltages (V>60 mV)
hVDAC3 C2-122A	YES	800-900 pS (*n* = 4)	No Voltage-dependence
hVDAC3 C8-122A	NO	350-400 pS (*n* = 8)	No Voltage-dependence
hVDAC3 C2-8-122A	YES	1100 pS (*n* = 11)	Tendency to close only at high applied voltages (≥80 mV)

### Mass spectrometry demonstrates that VDAC3 cysteines exist in different oxidation states

**Figure 5:**
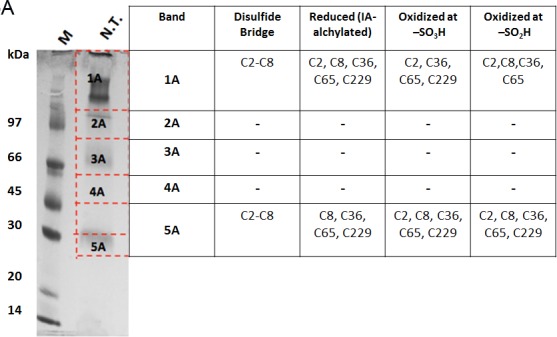

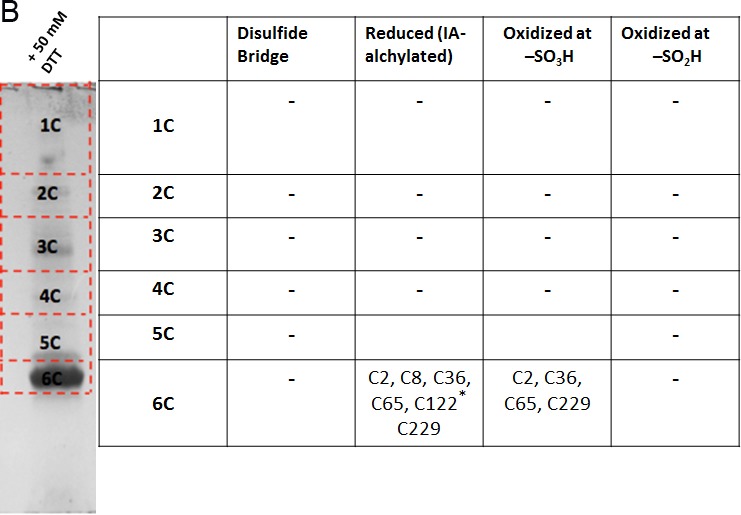

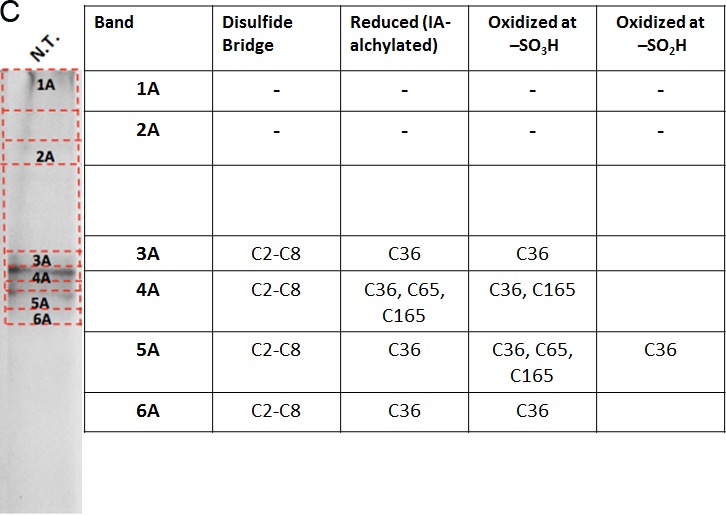

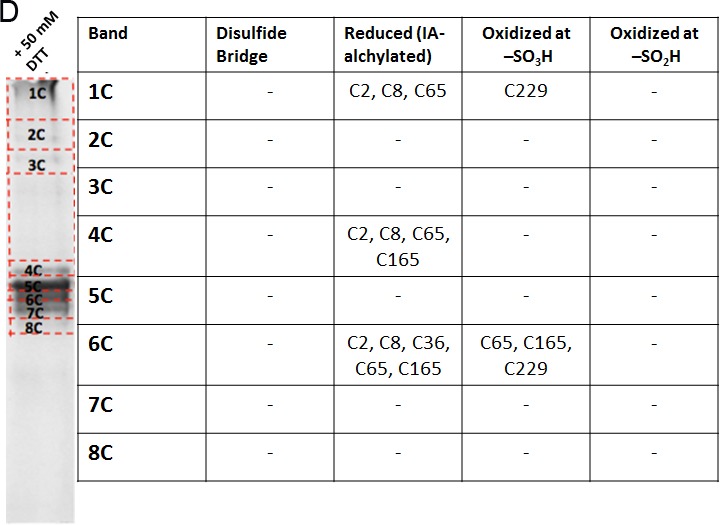
Mass spectrometry analysis reveals the presence of different redox patterns of VDAC3 cysteines in electrophoretic distinguishable protein bands Recombinant refolded hVDAC3 or rat liver mitochondria HTP pass-through were overloaded on 17.5% SDS-PAGE. Each separated band was cut out of the gel and the content was subject to Mass Spectrometry. The redox state of the tryptic peptides present in each band was detected and annotated in the corresponding Table. **A.** Non-treated recombinant hVDAC3 was loaded on a 12% SDS-PAGE and run without any reducing agent. Coomassie blue stained polypeptides were identified as band 1A to 5A. MW: standard Mr markers (Sigma). **B.** Recombinant hVDAC3 was reduced with 50 mM DTT and loaded on a 12% SDS-PAGE. Coomassie blue stained polypeptides were identified as bands 1C to 6C. (*) indicates that C122 was only found upon chymotrypsin digestion. **C.** Non-treated HTP eluate form rat liver mitochondria was loaded on SDS-PAGE and run without any reducing agent. Coomassie blue stained polypeptides were identified as band 1A to 6A. **D.** HTP eluate form rat liver mitochondria was reduced with 50 mM DTT and loaded on SDS-PAGE. Coomassie blue stained polypeptides were identified as band 1C to 8C.

The oxidation state of hVDAC3 cysteines was thoroughly investigated by mass spectrometry. The analysis was performed both on recombinant, refolded hVDAC3 and on VDAC3 purified from rat liver mitochondria. Recombinant hVDAC3 was overloaded on SDS-PAGE gels without any treatment (Figure 5A) or following reduction with DTT (Figure 5B). Stained bands were cut out and treated as described in Methods. A set of gel bands was not subjected to the reducing/blocking treatment prior to the addition of proteolytic enzymes in order to detect the cysteine(s) oxidized state(s) (including the putative presence of disulfide bridges). Another set of gel slices was reduced by DTT and alkylated with iodoacetamide to avoid further modifications during the detection procedure. The resulting peptides were detected by an Orbitrap Fusion™ Tribrid™ mass spectrometer and analyzed. The state of the cysteines that we were able to detect is summarized alongside the gel (Figure 5A-5B). Analysis of the untreated sample led to the identification of a disulfide bridge between the cysteine 2 and 8 in the N-terminal domain (Suppl. Figure 3A). The S-S bridge was present not only in the aggregated form of the protein (band 1A in Figure 5A) but also in the monomeric form (band 5A in Figure 5A). C2 and C8, together with C36, C65, and C229 were found also in different oxidation states: sulfonic, sulfinic and -SH reduced form. However, in hVDAC3 treated with DTT (Figure 5B), most of the protein was in the monomeric form, and the trace amounts found in the bands 1C-5C did not permit a reliable detection. In the band 6C, every cysteine was found in the reduced form, comprising C122 that was detected thanks to an alternative cleavage with chymotrypsin. Nevertheless, most of the cysteines were also found in the sulfonic acid state, an irreversible oxidation state that cannot be reduced by DTT treatment. Cysteines were then analyzed in VDAC3 prepared directly from mitochondrial extracts. Rat liver mitochondria were solubilized with Triton X-100 and chromatographed through a hydroxyapatite (HTP) column [34]. Since there is no established protocol for the purification of VDAC3 from tissues, the protein is enriched in the HTP eluate [35] that contains several electrophoretic bands in the range of 30-35 kDa [35]. The HTP eluate was either reduced or oxidized and run on a gel. Stained bands were cut out and treated for mass spectrometry analysis as reported above. It must be taken into account that rat VDAC3 has a seventh cysteine (Cys165), in addition to the six in human (Figure 1A). Figure 5C shows that in the untreated sample, VDAC3 was found in different gel bands in the 30-35 kDa area, most likely due to the different redox states of cysteines. Also in the mitochondria derived protein, the absence of a reducing treatment of the sample allowed the identification of a disulfide bridge between cysteine 2 and 8. Cysteine 36 was detected in all possible oxidative states (reduced, sulfonic and sulfinic), while C65 and C165 were found in both reduced and sulfonic states. Coverage of C229 was not obtained. In the reduced sample (Figure 5D), the disulfide bridge was obviously absent and cysteines 2, 8 and 36 were found only in the reduced form. C65 and C165 were found reduced, but also irreversibly oxidized and C229 was detected only in the sulfonic form (band 1C and 6C Figure 5D). The peptide containing Cys122 was never detected, possibly because part of a large tryptic peptide. Interestingly, the stained band at high molecular weight in SDS-PAGE (Figures 5D, band 1C) contains evidence of rat VDAC3 peptides: this means that VDAC3 can also aggregate in mitochondria. VDAC1 has earlier been shown to be able to form homomultimers/aggregates under certain conditions (e.g. [36]).

### Molecular dynamics analysis of cysteines 2-8 disulfide bridge in VDAC3

**Figure 6:**
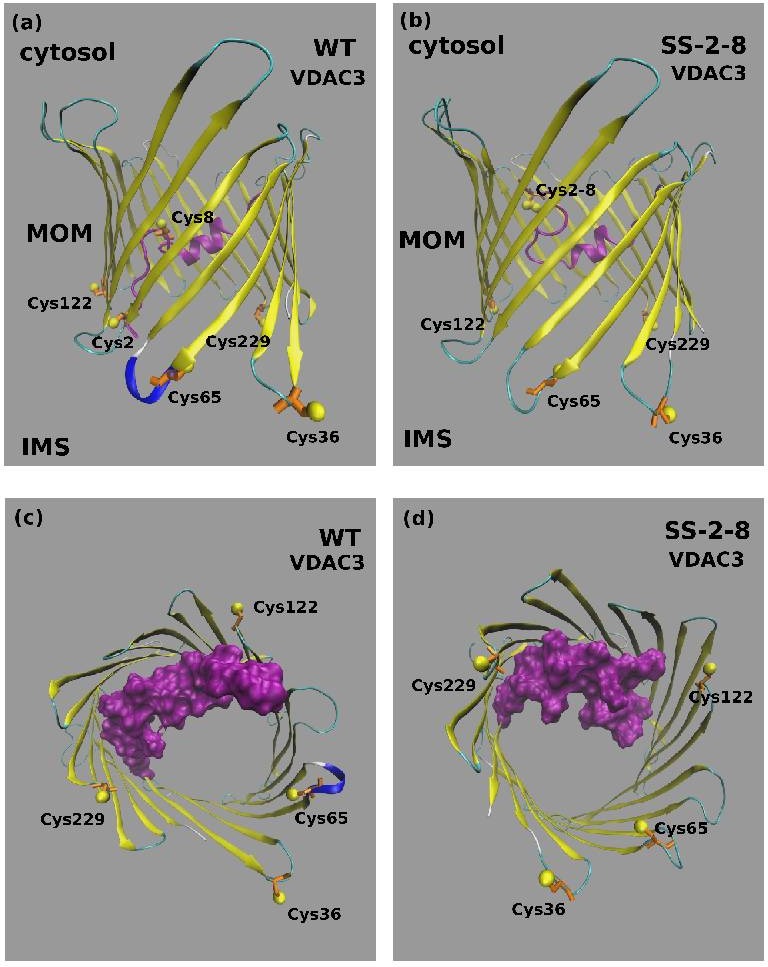
Molecular Dynamics model of VDAC3 containing the disulfide bond 2-8 **A.** Typical structure of the wild type VDAC3 pore and **B.** of the disulfide bonded variant Cys 2-8 (SS 2-8). The N-terminal tail corresponding to fragment 1-25 is shown in magenta. The cysteine residues are shown in a ball-and stick representation. **C.** Top view of the wild type VDAC3 channel and **D.** of the disulfide bonded variant Cys 2-8 (SS 2-8). The N-terminal tail corresponding to fragment 1-25 is shown in magenta with a surface filling representation that highlights the different degree of obstruction of the channel. The cysteine residues are shown in a ball-and stick representation.

A disulfide bridge can form in mitochondrial VDAC3. Suppl. Figure 3A-3B shows the spectra of representative tryptic peptides containing the disulfide bridge C2-8 both in the recombinant hVDAC3 and in the mitochondrial rat VDAC3. The VDAC3 disulfide bridge C2-8 was simulated by molecular dynamics (MD), to investigate the functional consequences of the bond. The structural features of the disulfide-bonded variant of the VDAC3 channel are illustrated in Figure 6, where the last frame of the fifth 100 ns production run is shown. A feature of the VDAC channel is that both the N-terminal and C-terminal point towards the same direction (positive z-axis in our simulations). In VDAC3 containing the disulfide bridge C2-8, the disulfide bends the N-tail over itself so that fragment 1-12 now runs downwards roughly parallel to segment 13-25 that is still directed upwards. In the MD simulation, the bend induced by the covalent disulfide bond causes the whole N-terminal to stay closely packed to the wall of the barrel (strands 3-9). The N-tail bending results in a certain level of obstruction of the channel. This can be better appreciated in Figure 6B, where a top view is shown. The steric hindrance of the VDAC3 channel in the disulfide bonded species was quantified through a calculation of the solvent accessible surface area (ASA). From the profile of ASA (Suppl. Figure 4), a decrease occurs in the lower region of the pore in the case of VDAC3 containing the disulfide bridge 2-8. It can be noted that the drop in the accessible surface caused by the formation of the disulfide bridge 2-8 is small (about 12%). Assuming that this model is coherent with the physiological state of the pore, the ion current in the S-S bridged VDAC3 should be only slightly lower with respect to the form with the same cysteines in reduced state. Concerning the possible pathways for cations and anions, we based on a previous work [25] where we found two possible paths for chloride ions, one close to the N-terminal helix, the second one near the barrel and, on the other hand, only one path for potassium. The presence of the C2-8 disulfide bond should strongly perturbate the chloride path close to the N-terminal domain. Chlorides, however, can still cross the channel through the alternative path.

### Chemico-physical measurements reveal that cysteine 8 stabilizes hVDAC3

**Figure 7:**
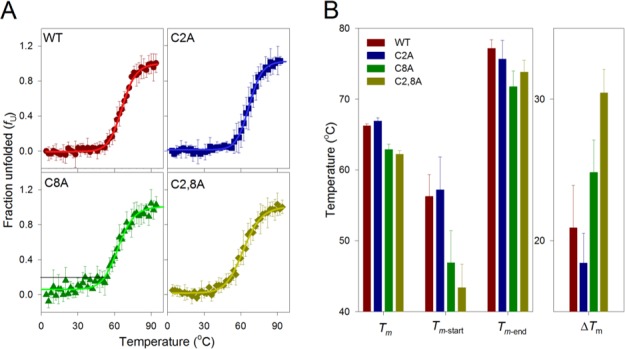
Thermal denaturation studies of hVDAC3 **A.** Change in secondary structure content with increase in temperature plotted as fraction unfolded (*f*u) and fitted to a two state model (solid lines). The unfolding profiles for hVDAC3 WT (red circle), C2A (blue square), C8A (green triangle) and C2,8A (olive diamond) mutants are shown in the different panels. The error bars are generated from an average of 3-4 independent measurements. Black line directed to the *y*-intercept in the C8A panel highlights the drifting baseline, which indicates a marginal loss in the secondary structure content by ∼20%, before onset of the actual unfolding. **B.** Thermal parameters (*T_m_*, *T_m-start_*, *T_m-end_* and Δ*T_m_*) were calculated (see Suppl Fig. 5 for the calculation) from *f*u plots, and the contribution of cysteine residues to the protein stability was compared. The first set of bars show the comparative view of thermal midpoint (*T_m_*) values, indicating higher stability of the WT and C2A, with respect to the cysteine mutants C8A and C2,8A. The *T_m-start_* values corresponding to the initiation of unfolding also show that C8A and C2,8A unfold at lower temperatures, compared to WT and C2A. Similarly, the *T_m-end_* value is highest for WT and C2A, again inferring that end point is reached at higher temperatures for these mutants. The difference between *T_m-end_* and *Tm*-start (Δ*T_m_*) reflects the cooperativity of the unfolding process. WT and C2A show lower Δ*T_m_* values, suggesting that unfolding is highly cooperative in these mutants and is least cooperative for the double mutant C2,8A. Each data set represents an average of 3-4 independent experiments. A similar colour scheme is retained across all the panels.

Commensurate with its predicted structure, hVDAC3 exhibits a characteristic far-UV circular dichroism (CD) profile of a β-barrel protein (Suppl. Figure 5). The mutation of cysteine residues does not largely affect the overall structure of the protein (Suppl. Figure 5). Therefore, we examined the stability of hVDAC3 to unfold by heat, by monitoring changes in secondary structure using reported CD methods [37]. When we denatured transmembrane β-barrels such as VDAC by heating, protein unfolding was irreversible. Such irreversible nature is a biophysical characteristic common for proteins that are under kinetic control [38]. Heating weakens protein interaction with its surrounding lipid environment, and causes the protein to unfold. Once unfolded, aggregation ensues immediately - this process buries hydrophobic segments of the protein from the aqueous environment. Such non-specific protein aggregation differs from the oligomerization of VDACs, by the nature of the pathway. The folded VDAC mediates protein oligomerization in the membrane in a controlled and reversible manner [39]. However, irreversible aggregation that we obtained in our thermal denaturation measurements is non-specific, is nucleated by the unfolded protein, occurs outside the membrane and is irreversible [40]. In our experiments, hVDAC3 and its cysteine mutants exhibited irreversible thermal denaturation, with coupled unfolding and aggregation events. In 1% LDAO, the unfolding is cooperative for all the proteins, with the folded and aggregated states linked by a two-state process (Figure 7). A closer examination of the unfolding data reveals that in hVDAC3-C8A, the pre-transition baseline suggests that the protein undergoes a marginal loss in secondary structure by ∼20%, before the onset of the two-state transition. This indicates that C8 may play an important role in hVDAC3 stabilization. We derived the thermal unfolding parameters *T_m-start_*, *T_m_* and *T_m-end_* for all the proteins. This is summarized in Suppl. Figure 6. The overall behavior of the C2A mutant was similar to that of native VDAC3, indicating that this cysteine may be dispensable for the stability of the barrel. However, upon the mutation of C8, in hVDAC3 C8A, we observed a reduction in the *T_m_* by ∼4°C. This is further manifested in the lowered *T_m-start_* by ∼10°C, resulting in a considerable difference in Δ*T_m_*. This data suggests that C8 plays a key role in structuring hVDAC3. Indeed, a close examination of the far-UV CD profiles reveals that the secondary structure content was marginally lowered for C8A (Suppl. Figure 5). The C2,8A mutant is further destabilized, when compared with hVDAC3 C8A. We, therefore, surmise that the cysteine residues present at the N-terminal helix of hVDAC3 are important to stabilize the barrel. Thus, their oxidation to form a disulfide bridge is destabilizing and possibly contributes to a conformational change of the protein.

## DISCUSSION

Cell requires perfectly functioning mitochondria. Nevertheless, the organelle is the source of redox species. The accumulation in mitochondria of oxidative agents can damage the organelle and cause in turn problems to the whole cell. An out-of-control ROS load in mitochondria must be signaled to the cell and an organelle heavily loaded with ROS must be evidenced and destroyed, if ineffective. The front-runner for this function is the mitochondrial outer membrane, as the border of the organelle towards the rest of the cell. VDACs are the most abundant protein spanning the MOM and thus connecting the IMS to the cytosol. The mitochondrial intermembrane space (IMS) has physicochemical properties distinct from the matrix and the two membranes: the pH is more acidic than cytosol by 0.2-0.7 units [25, 41] and the glutathione redox buffer is more oxidizing than in cytosol and in the matrix [42]. This composition produces an oxidizing environment that, in the cell, is exceeded only by the ER lumen. In addition, IMS is characterized by the abundance of ROS poured here mainly by Complex III [43, 44] but also by Complex II [45] and by the activity of MAO [24]. A delicate equilibrium, involving the presence of detoxifying enzymes like SOD1, permits the maintenance of “redox homeostasis”. ROS released from the IMS serve as signaling molecules to induce gene expression in the nucleus [46]. Among ROS, O_2_- superoxide is very active although has a short life, while hydrogen peroxide (H_2_O_2_) is a permeable signaling agent able to oxidize cysteines to disulfides [47].

We have previously proposed the transmembrane topology of VDAC1 [22]. Based on this model, it has been predicted that four out of six cysteines of hVDAC3 are exposed toward the IMS and the remaining two are in N-terminal moiety. In this scenario the VDAC3 cysteines are likely candidates to oxidation by ROS in the IMS. We found indeed that cysteines in VDAC3 can bear various oxidation states and a disulfide bridge between cysteine 2 and 8 was also proven. Evidence for “overoxidation” of thiols, meaning the irreversible oxidation of a cysteine thiol to sulfinic or sulfonic acid [48] was reported in other works [44].

A very recent work, basically simultaneous to the present one, has proposed that cysteine oxidation and the formation of a disulfide bridge influence VDAC3 channel gating [33]. In their paper Okazaki et al reported the effect of recombinant VDAC3 reduction by DTT and studied some Cys to Ala mutagenized VDAC3. Both groups came to the same conclusion, i.e. that cysteines are relevant for the pore-function. Okazaki and colleagues hypothesized that the formation of the disulfide bridge between residues 2 and 122 can influence the gating of the pore, favouring the closed state [33]. Our mass spectrometry analysis determined, instead, that a disulfide bridge is formed between Cys 2 and Cys8 but not between Cys 2 and Cys 122. Since the former disulfide bridge affects the N-terminal region, its relevance for the conductance of the pore appears to be limited, as shown by MD. On the other hand, the partial hindrance of the pore may have influence on the interaction with tubulin, a physiological inhibitor of the pore that plugs into its cavity [49]. It cannot be excluded that other disulfide bridges can form between other cysteines pairs: these events must not be based only on physical proximity but likely depend on other chemico-physical properties of the surrounding structure as well. Unfortunately, experimental evidence to support this latter hypothesis is still missing. A relevant difference between our analysis and the work by Okazaki et al [33] is the observation reported here that VDAC3 cysteines may be found in several and variable oxidation states in addition to the disulfide bridge: this feature made the whole analysis of the protein function more complicated than expected but, importantly, it underlines a so-far unknown feature of VDACs which occurs also *in vivo*, as found in rat liver mitochondria.

As a counter-proof of the importance of redox states of VDAC3 cysteines, the removal of selected cysteines reduces the dynamic range of oxidation states and had a clear impact on the function. These conclusions are based on the clear demonstration of changes in the electrophoretic pattern of cysteine mutants compared to hVDAC3, as discussed also in [33]. Even more interesting is the effect of such deletions on VDAC3 gating. The electrophysiological data reported here show that deletion of C2 alone or together with C8 and/or C122 enable VDAC3 to insert more easily into an artificial membrane and to adopt a higher conductance state than the wtVDAC3. This result is in accord with the recent finding that the deletion of the Cys2 and 8 together, or the Cys 122 alone, changed the gating features and the voltage dependence of VDAC3 leading to a behavior resembling the characteristics of the other isoforms [33]. These three residues can indeed be considered the most relevant ones for the control of gating and determination of conductance of the VDAC3 pore. Importantly we have demonstrated here that there is a correlation between the gating properties of the recombinant VDAC3 mutants and their ability to complement the yeast growth in Δ*porin1* cell, since the mutants where Cys8 was mutagenized in alanine (C8A, C2,8A (partially), C2,122A and C2,8,122A) in general showed an higher activity than the wtVDAC3 when the cells grew on non fermentable carbon source.

Furthermore, C2 and C8 may be involved in VDAC oligomerization upon extrusion of the N-terminal domain, as evidenced by the fact that the simultaneous absence of both residues or of one of them along with C122 abolishes aggregation *in vitro*. Previous works suggested that VDAC1 N-terminal domain can be exposed, or at least be very accessible, from outside the pore, and can stimulate the formation of VDAC super-assemblies [50, 51, 21]. When a cysteine was introduced in the VDAC1 N-terminal sequence and the α-helix motif was destroyed by the introduction of proline, evidence of VDAC1 oligomerization through intermolecular disulfide formation was presented [51].

Interestingly, human VDAC2 also possesses higher cysteine content than hVDAC3 [37]. Functionally, however, VDAC2 can show larger conductance and different gating characteristics with respect to VDAC3 [18]. Recent electrophysiology measurements show that the deletion of all nine cysteines of VDAC2 has a marginal effect on channel function *in vitro* [40]. Furthermore hVDAC2 and hVDAC3 share four common cysteines (2, 36, 65 and 122; numbering based on hVDAC3), of which we observe athat C2 and C122 are important in VDAC3.

Evidence of VDAC3 as a preferred target of mitochondrial ROS generated by the complex III has recently come from a redox DIGE (two-dimensional fluorescence difference gel electrophoresis) proteomic study performed on intact and highly coupled rat heart mitochondria [44]. This method is based on differential thiol labeling with fluorescent CyDye maleimides and detects reversible oxidations of protein thiols. The authors convincingly showed that proteins of the intermembrane space, such as the mitochondrial import inner membrane translocase subunit TIM50, or the outer membrane protein VDAC3 were heavily targeted by complex III-derived ROS. VDAC3 featured a strikingly high increase in thiol oxidation by ROS. It is very relevant to our discussion that only VDAC3 was found oxidized but not VDAC2, despite the high number of cysteines in the latter protein. The authors concluded that “the pronounced selectivity by which some proteins were oxidized qualifies them as highly useful potential biomarkers to monitor the generation of mitochondrial ROS at specific sites and under defined conditions”, an implicit consideration about a relevant role of VDAC3 as redox sensor [44].

The question arises what is the function of VDAC3 cysteine oxidations in a cellular context? Besides establishing the protein fold, the functional meaning of disulfides is not known. Mitochondrial inner membrane (MIM) proteins like subunits of Complex I, the Rieske protein in complex III, subunits of the TIM translocases, and members of the mitochondrial carrier family [52] contain conserved cysteines facing the IMS. The oxidation of VDAC3 cysteines as described in this work could be the result of a balance between cysteine oxidation and reduction reactions taking place in the IMS. VDAC3 oxidations could also be used to keep track of the redox level in the IMS. Cysteine oxidation can be counterbalanced by the redox restoring systems like thioredoxins or glutathione. Nevertheless, the oxidation to sulfonic acid is irreversible and leads to a permanently modified (damaged) protein that can undergo conformational changes or degradation. Interestingly, in a proteomic survey of VDAC3-interacting proteins [53], there were found proteins able to act as transport proteins as serpin-6 or Ter-ATPase (VCP) (that transfers membranes from the ER, in close contact with the MOM, to the Golgi apparatus *via* specific transition vesicles), indicating the propensity of VDAC3 to be destroyed when heavily damaged (oxidized).

In conclusion, we hypothesize that VDAC3 may not function only as a simple channel within the cell because of its high content of oxidation sensible cysteine residues, despite its general structure is possibly close to the other VDAC isoforms. Further studies will be needed to explore the particular role VDAC3 may have in redox signaling and response mechanisms to oxidative stress.

## MATERIALS AND METHODS

### Cloning and mutagenesis

cDNAs for hVDAC1, hVDAC3 and hN1-VDAC3 were cloned in pET21a vector (Novagen) with a C-terminal 6xHis-tag, as reported in [19, 21]. Mutagenesis was performed on hVDAC3 sequence by the QuickChange Site-Directed Mutagenesis kit (Stratagene).

Specific couples of primers, designed to replace cysteine residues (C) with alanines (A), are reported as follow: C2A(5′-GGAGATATACATATGGCTAACACACCAACGTAC-3′,5′-GTACGTTGGTGTGTTAGCCATATGTATATCTCC-3′); C8AACACACCAACGTACGCTGACCTAGGAAAGGCT-3′,5′-AGCCTTTCCTAGGTCAGCGTACGTTGGTGTGTT-3′); C122A(5′-TCCTATAAACGGGATGCTTTTAGTGTTGGCAGT-3′,5′-ACTGCCAACACTAAAAGCATCCCGTTTATAGGA-3′). After confirming the single mutations by DNA sequencing, further PCR rounds were performed to create the double mutants hVDAC3 C2,8A, C2,122A, C8,122A, and the triple mutant hVDAC3 C2,8,122A. cDNAs for hVDAC1, hVDAC3, hN1-VDAC3 and hVDAC3 cys-mutants were also cloned in pYX212 vector, as previously described [20]. All the constructs were confirmed by DNA sequencing.

### Expression, purification and refolding of 6xHis-tagged proteins

*Escherichia coli* BL21(DE3) cells were transformed with pET21a containing the native or mutagenized hVDAC genes. Proteins expression was performed by addition of 1 mM isopropyl-β-D-thiogalactopyranoside (IPTG, Sigma) at an optical density (λ = 595 nm) of ∼0.6 at 37°C for 3 h, as reported in [21]. Bacterial cell were resuspended in buffer B (8 M urea, phosphate buffer, pH 8.0) and shaked overnight at 4°C. After pelleting cell debris by centrifugation, the clear lysate was loaded onto a Ni-NTA agarose (Qiagen) packed column pre-equilibrated with 10 column volumes of buffer B. The column was then washed twice with 5 volumes of buffer C (8 M urea, phosphate buffer, pH 6.2) and the purified proteins were eluted with 5 volumes of buffer E (8 M urea, phosphate buffer, pH 3.5). The denatured protein mixture was added drop-wise to a refolding buffer (25 mM Tris, 100 mM NaCl, 1 mM EDTA, 1% (v/v) lauryldimethylamine-oxide (LDAO, Sigma), pH 7.0) in order to obtain a ten-fold dilution of the urea concentration, and was gently stirred overnight at 4°C. The protein solution was dialyzed against 100 volumes of a dialysis buffer (25 mM Tris, 1 mM EDTA, 0.1% LDAO, pH 7.0) with Thermo Scientific Slide-A-Lyzer Dialysis Cassettes (3.5 K MWCO) two times for 2 h and one time for 1 day at 4°C. All the steps of the refolding procedure were carried out without adding any reducing agent. The protein purity was verified by SDS-PAGE and Comassie staining. Purified samples were stored at −20°C.

### Preparation of VDAC3 enriched fractions from rat liver mitochondria

Rat liver mitochondria were prepared by standard procedure [34]. 25 mg of rat liver mitochondria were solubilized for 20 min on ice with 5 ml of buffer A (3% Triton X-100, 10 mM TrisHCl, 1 mM EDTA pH 7.0) and then centrifuged at 17,400 x *g* for 30 min at 4°C. The supernatant was loaded onto 5 g of dry hydroxyapatite (Bio-Gel HTP, Biorad) packed in a glass Econo-column 2.5 × 30 cm (Biorad). The column was eluted at 4°C with 3 ml of buffer A and five fraction of 0.6 ml were collected [34]. Samples were stored at −20°C.

### Electrophoretic analysis

Proteins electrophoretic techniques are reported in the Supplementary Materials.

### Electrophysiological analysis

Refolded hVDAC3 wild type and mutants were reconstituted into a PLB (planar lipid bilayer) as described previously [19]. Bilayers of approx. 150-200 pF capacity were prepared using purified soybean asolectin. The standard experimental medium contained 1 M KCl, 10 mM Hepes/pH 7.2 (set using KOH). Control experiments with empty membrane or with detergents used for the purification showed no activity. All voltages reported are those of the *cis* chamber, zero being assigned by convention to the *trans* (grounded) side. Currents are considered as positive when carried by cations flowing from the *cis* to the *trans* compartment. Data were acquired using a Bilayer Clamp amplifier (Warner Instruments, USA) at 100 μs/point, filtered at 300 Hz and analyzed offline using the pClamp program set (Axon Instruments, Union City, CA, USA). Histograms were fitted using Origin7.5 program set. Leak was not subtracted.

### Yeast strains and viability spot assay

Wild type *Saccharomyces cerevisiae* strain BY4742 [MAT a, his3Δ1, leu2Δ0, lys2Δ0, ura3Δ0] and isogenic porin-depleted mutant *Δpor1* [MATa, his3Δ1, leu2Δ0, lys2Δ0, ura3Δ0, por1::kanMX4] were obtained from EUROSCARF (Frankfurt, Germany) as already reported [20]. *Δpor1* strain was used for heterologous expression of native or mutant hVDACs; BY4742 was used as reference. Yeast strains were grown on rich medium YP (1% yeast extract, 2% peptone) supplemented with 2% glucose (YPD) or 2% glycerol (YPY) and minimal medium (0.67% yeast nitrogen base) containing 2% glucose (SD) or 2% glycerol (SY) supplemented with 10 μg/ml of the appropriate nutritional requirements according to the genotype of the strains. Agar (2%) was added for solid plates. For the viability spot assay, cell suspensions at the concentration of 10^8^ cells/mL were transferred in microtiter plates, serially ten-fold diluted and spotted onto YPD and YPY plates. Incubation was performed at 28°C and 37°C for three-five days before recording.

### Mass spectrometry analysis

Mass spectrometry data were acquired on an Orbitrap Fusion Tribrid (Q-OT-qIT) mass spectrometer (ThermoFisher Scientific, Bremen, Germany) equipped with a ThermoFisher Scientific Dionex UltiMate 3000 RSLCnano system (Sunnyvale, CA). Full details are in the Supplementary Materials.

### Thermal denaturation experiments

Wild type and mutants hVDAC3 cloned in pET21a were expressed as inclusion bodies in *E. coli C41* cells. Protein purification was performed using reported methods [54]. Protein refolding was achieved by removing guanidine hydrochloride according to the protocol described previously for hVDAC2 [40]. The secondary structure content of refolded hVDAC3 was determined using far-UV circular dichroism (CD) spectropolarimetry on a Peltier-controlled JASCO J-815 instrument. Further details are presented in Supplementary Materials.

### Molecular dynamics simulations

The Molecular Dynamics simulations of the hVDAC3 channel with and without a disulfide bond in position 2-8 were run on a homology model built using the mouse VDAC1 as template as detailed in [26]. Technical description of the procedure is reported in the Supplementary Materials.

## SUPPLEMENTARY MATERIAL FIGURES



## References

[1] Messina A, Reina S, Guarino F, De Pinto V (2012). VDAC isoforms in mammals. Biochim. Biophys. Acta.

[2] Colombini M (1980). Structure and mode of action of a voltage dependent anion-selective channel (VDAC) located in the outer mitochondrial membrane. Ann. N.Y. Acad. Sci..

[3] Benz R (1994). Permeation of hydrophilic solutes through mitochondrial outer membranes: review on mitochondrial porins. Biochim. Biophys. Acta.

[4] Rostovtseva T, Colombini M (1997). VDAC channels mediate and gate the flow of ATP: implications for the regulation of mitochondrial function. Biophys. J.

[5] Kroemer G, Galluzzi L, Brenner C (2007). Mitochondrial Membrane Permeabilization in Cell Death. Physiol. Rev.

[6] Tomasello F, Messina A, Lartigue L, Schembri L, Medina C, Reina S, Thoraval D, Crouzet M, Ichas F, De Pinto V, De Giorgi F (2009). Outer membrane VDAC1 controls permeability transition of the inner mitochondrial membrane in cellulo durings stress-induced apoptosis. Cell Res.

[7] De Stefani D, Bononi A, Romagnoli A, Messina A, De Pinto V, Pinton P, Rizzuto R (2012). VDAC1 selectively transfers apoptotic Ca(2+) signals to mitochondria. Cell Death Differ.

[8] Shoshan-Barmatz V, Mizrachi D (2012). VDAC1: from structure to cancer therapy. Front. Oncol.

[9] Lemasters JJ, Holmuhamedov E (2006). Voltage-dependent anion channel (VDAC) as mitochondrial governator - thinking outside the box. Biochim. Biophys. Acta.

[10] Huizing M, Ruitenbeek W, Thinnes F, De Pinto V (1994). Deficiency of the Voltage-Dependent Anion Channel (VDAC): a novel cause of mitochondrial myopathies. Lancet.

[11] Hiller S, Garces RG, Malia TJ, Orekhov VY, Colombini M, Wagner G (2008). Solution structure of the integral human membrane protein VDAC-1 in detergent micelles. Science.

[12] Ujwal R, Cascio D, Colletier JP, Faham S, Zhang J, Toro L, Ping P, Abramson J (2008). The crystal structure of mouse VDAC1 at 2.3 A resolution reveals mechanistic insights into metabolite gating. Proc. Natl. Acad. Sci. U.S.A.

[13] Bayrhuber M, Meins T, Habeck M, Becker S, Giller K, Villinger S, Vonrhein C, Griesinger C, Zweckstetter M, Zeth K (2008). Structure of the human voltage-dependent anion channel. Proc. Natl. Acad. Sci. U.S.A.

[14] Schredelseker J, Paz A, López CJ, Altenbach C, Leung CS, Drexler MK, Chen JN, Hubbell WL, Abramson J (2014). High resolution structure and double electron-electron resonance of the zebrafish voltage-dependent anion channel 2 reveal an oligomeric population. J. Biol. Chem.

[15] Hiller S, Wagner G (2009). The role of solution NMR in the structure determinations of VDAC-1 and other membrane proteins. Curr. Opin. Struct. Biol.

[16] Sampson MJ, Lovell RS, Craigen WJ (1997). The murine voltage-dependent anion channel gene family. Conserved structure and function. J. Biol. Chem.

[17] De Pinto V, Guarino F, Guarnera A, Messina A, Reina S, Tomasello FM, Palermo V, Mazzoni C (2010). Characterization of human VDAC isoforms: a peculiar function for VDAC3?. Biochim. Biophys. Acta.

[18] Xu X, Decker W, Sampson MJ, Craigen WJ, Colombini M (1999). Mouse VDAC isoforms expressed in yeast: channel properties and their roles in mitochondrial outer membrane permeability. J. Membr. Biol.

[19] Checchetto V, Reina S, Magrì A, Szabò I, De Pinto V (2014). Recombinant human Voltage Dependent Anion selective Channel isoform 3 (VDAC3) forms pores with a very small conductance. Cell Physiol. Biochem.

[20] Reina S, Palermo V, Guarnera A, Guarino F, Messina A, Mazzoni C, De Pinto V (2010). Swapping of the N-terminus of VDAC1 with VDAC3 restores full activity of the channel and confers anti-aging features to the cell. FEBS Lett.

[21] Reina S, Magrì A, Lolicato M, Guarino F, Impellizzeri A, Maier E, Benz R, Ceccarelli M, De Pinto V, Messina A (2013). Deletion of β-strands 9 and 10 converts VDAC1 voltage-dependence in an asymmetrical process. Biochim. Biophys. Acta.

[22] Tomasello F, Guarino F, Reina S, Messina A, De Pinto V (2013). The voltage-dependent anion selective channel 1 (VDAC1) topography in the mitochondrial outer membrane as detected in intact cell. PLoS One.

[23] Cochemé HM, Murphy MP (2008). Complex I is the major site of mitochondrial superoxide production by Paraquat. J. Biol. Chem.

[24] Kaludercic N, Mialet-Perez J, Paolocci N, Parini A, Di Lisa F (2014). Monoamine oxidases as sources of oxidants in the heart. J. Mol. Cell. Cardiol.

[25] Porcelli AM, Ghelli A, Zanna C, Pinton P, Rizzuto R, Rugolo M (2005). pH difference across the outer mitochondrial membrane measured with a green fluorescent protein mutant. Biochem Biophys Res Commun.

[26] Amodeo GF, Scorciapino MA, Messina A, De Pinto V, Ceccarelli M (2014). Charged Residues Distribution Modulates Selectivity of the Open State of Human Isoforms of the Voltage Dependent Anion-Selective Channel. PLoS One.

[27] Yamamoto T, Yamada A, Watanabe M, Yoshimura Y, Yamazaki N, Yoshimura Y, Yamauchi T, Kataoka M, Nagata T, Terada H, Shinohara Y (2006). VDAC1, having a shorter N-terminus than VDAC2 but showing the same migration in an SDS-polyacrylamide gel, is the predominant form expressed in mitochondria of various tissues. J Proteome Res.

[28] Girard PM, Graindorge D, Smirnova V, Rigolet P, Francesconi S, Scanlon S, Sage E (2013). Oxidative stress in mammalian cells impinges on the cysteines redox state of human XRCC3 protein and on its cellular localization. PLoS One.

[29] Pereira C, Chaves S, Alves S, Salin B, Camougrand N, Manon S, Sousa MJ, Côrte-Real M (2010). Mitochondrial degradation in acetic acid-induced yeast apoptosis: the role of Pep4 and the ADP/ATP carrier. Mol. Microbiol.

[30] Pereira C, Camougrand N, Manon S, Sousa MJ, Côrte-Real M (2007). ADP/ATP carrier is required for mitochondrial outer membrane permeabilization and cytochrome c release in yeast apoptosis. Mol. Microbiol.

[31] Colombini M (2012). VDAC structure, selectivity, and dynamics. Biochim. Biophys. Acta.

[32] Szabò I, Zoratti M (2014). Mitochondrial channels: ion fluxes and more. Physiol. Rev.

[33] Okazaki M, Kurabayashi K, Asanuma M, Saito Y, Dodo K, Sodeoka M (2015). VDAC3 gating is activated by suppression of disulfide-bond formation between the N-terminal region and the bottom of the pore. Biochim Biophys Acta.

[34] De Pinto V, Prezioso G, Palmieri F (1987). A simple and rapid method for the purification of the mitochondrial porin from mammalian tissues. Biochim. Biophys. Acta.

[35] Yamamoto T, Tamaki H, Katsuda C, Nakatani K, Terauchi S, Terada H, Shinohara Y (2013). Molecular basis of interactions between mitochondrial proteins and hydroxyapatite in the presence of Triton X-100, as revealed by proteomic and recombinant techniques. J. Chromatogr. A.

[36] Shoshan-Barmatz V, De Pinto V, Zweckstetter M, Raviv Z, Keinan N (2010). VDAC, a multifunctional mitochondrial protein regulating cell life and death. Mol. Aspects Med.

[37] Maurya SR, Mahalakshmi R (2013). Modulation of human mitochondrial voltage-dependent anion channel 2 (hVDAC-2) structural stability by cysteine-assisted barrel-lipid interactions. J. Biol. Chem.

[38] Maurya SR, Mahalakshmi R (2014). Influence of protein-micelle ratios and cysteine residues on the kinetic stability and unfolding rates of human mitochondrial VDAC-2. PLoS One.

[39] Raschle T, Hiller S, Yu TY, Rice AJ, Walz T, Wagner G (2009). Structural and functional characterization of the integral membrane protein VDAC-1 in lipid bilayer nanodiscs. J Am Chem Soc.

[40] Maurya SR, Mahalakshmi R (2015). N-helix and cysteines inter-regulate human mitochondrial VDAC-2 function and biochemistry. J Biol Chem.

[41] Cortese JD, Voglino AL, Hackenbrock CR (1992). The ionic strength of the intermembrane space of intact mitochondria is not affected by the pH or volume of the intermembrane space. Biochim. Biophys. Acta.

[42] Hu J, Dong L, Outten C.E. (2008). The redox environment in the mitochondrial intermembrane space is maintained separately from the cytosol and matrix. J. Biol. Chem.

[43] O-Uchi J, Ryu SY, Jhun BS, Hurst S, Sheu SS (2014). Mitochondrial ion channels/transporters as sensors and regulators of cellular redox signaling. Antioxid. Redox Signal.

[44] Bleier L, Wittig I, Heide H, Steger M, Brandt U, Dröse S (2015). Generator-specific targets of mitochondrial reactive oxygen species. Free Radic Biol Med.

[45] Orr AL, Quinlan CL, Perevoshchikova IV, Brand MD (2012). A refined analysis of superoxide production by mitochondrial sn-glycerol 3-phosphate dehydrogenase. J. Biol. Chem.

[46] Storz P (2007). Mitochondrial ROS: radical detoxification, mediated by protein kinase D. Trends Cell Biol.

[47] Zhang L, Xu H, Chen CL, Green-Church KB, Freitas MA, Chen YR (2008). Mass spectrometry profiles superoxide induced intramolecular disulfide in the FMN-binding subunit of mitochondrial Complex I. J. Am. Soc. Mass Spectrom.

[48] Winterbourn CC, Hampton MB (2008). Thiol chemistry and specificity in redox signaling. Free Radic. Biol. Med.

[49] Rostovtseva TK, Sheldon KL, Hassanzadeh E, Monge C, Saks V, Bezrukov SM, Sackett DL (2008). Tubulin binding blocks mitochondrial voltage-dependent anion channel and regulates respiration. Proc. Natl. Acad. Sci. U.S.A.

[50] Guo XW, Smith PR, Cognon B, D'Arcangelis D, Dolginova E, Mannella CA (1995). Molecular Design of the Voltage-Dependent, Anion-Selective Channel in the Mitochondrial Outer Membrane. J. Struct. Biol.

[51] Geula S, Ben-Hail D, Shoshan-Barmatz V (2012). Structure-based analysis of VDAC1: N-terminus location, translocation, channel gating and association with anti-apoptotic proteins. Biochem. J.

[52] Riemer J, Bulleid N, Herrmann JM (2009). Disulfide formation in the ER and mitochondria: two solutions to a common process. Science.

[53] Messina A, Reina S, Guarino F, Magrì A, Tomasello F, Clark RE, Ramsay RR, De Pinto V (2014). Live cell interactome of the human voltage dependent anion channel 3 (VDAC3) revealed in HeLa cells by affinity purification tag technique. Mol. Biosyst.

