# Supplementary material for: VDAC3 as a sensor of oxidative state of the intermembrane space of mitochondria: the putative role of cysteine residue modifications
Source: Oncotarget. 2016 Jan 8;7(3):2249–68. doi: 10.18632/oncotarget.6850 (PMC4823033; doi:10.18632/oncotarget.6850)
Supplement: Supplementary file 1 [file oncotarget-07-2249-s001.pdf]

VDAC3 as a sensor of oxidative state of the intermembrane space of mitochondria: the putative role of cysteine residue modifications

Supplementary Material

Electrophoretic analysis

Proteins electrophoretic patterns were analyzed after a 30 min incubation at 4°C of 10-20 µg of recombinant and refolded hVDAC with 1 mM diammide or 50 mM DTT. Samples were then precipitated with 9 volume of cold acetone and centrifuged for 20 min at 17,500 x *g*. Pellets were resuspended in NuPAGE LDS sample buffer 1 X (Invitrogen) without reducing agents and loaded on a 12% NuPAGE Novex gel (Invitrogen) together with a not treated control. Runs were performed with MES Running Buffer 1X. For mass spectrometry analysis, HTP eluates from rat liver mitochondria were overloaded on a 17% polyacrylamide gel with acrylamide-bis acrylamide ratio of 30:0.2 [S1]. Wild type and mutants hVDAC3 were overloaded on a 12% SDS-PAGE with acrylamide-bis acrylamide ratio of 30:0.2. Protein fractions were precipitated with 9 volumes of cold acetone for 30 min and then solubilized in SDS sample buffer (4% SDS, 20% glycerol, 0.004% bromphenol blue, 0.125 M Tris HCl, pH 6.8) without reducing agents. Protein bands were always visualized by Coomassie Blue staining.

Mass spectrometry analysis

In-gel digestion of 1D-SDS-PAGE bands. Three different experimental procedures were performed. In the first procedure, after SDS-PAGE separation, protein bands were manually cut in small pieces and subjected to the standard procedure of reduction and alkylation, followed by in-gel digestion using modified porcine trypsin or chymotrypsin [S2]. The recovered peptides were then lyophilized. The second procedure, aimed to the detection of disulfide bridges, was identical to the previous one, except that the reduction and alkylation steps were omitted. Finally, in the third procedure, which was carried out in order to confirm the presence of cysteines in the reduced form, the alkylation step was performed directly on the band pieces, omitting the reduction step.

Liquid chromatography and tandem mass spectrometry (LC–MS/MS) analysis.
Mass spectrometry data were acquired on an Orbitrap Fusion Tribrid (Q-OT-qIT) mass spectrometer (ThermoFisher Scientific, Bremen, Germany) equipped with a ThermoFisher Scientific Dionex UltiMate 3000 RSLCnano system (Sunnyvale, CA). Samples were reconstituted in 30 µL of 1% FA aqueous solution and 1 µL was loaded onto an Acclaim®Nano Trap C18 column (100 µm i.d. × 2 cm, 5 µm particle size, 100 Å). After washing the trapping column with solvent A (H_2O/CH_3CN, 98/2 + 0.1% FA) for 3 min at a flow rate of 7 µL/min, the peptides were eluted from the trapping column onto an PepMap® RSLC C18 Easy-spray column (75 µm i.d. × 15 cm, 3µm particle size, 100 Å). Peptides were separated at a flow rate of 300 nL/min at 40 °C with a linear gradient of solvent B (CH_3CN + 0.1% FA) in A from 1% to 60% over 30 min. Eluted peptides were ionized by a nanospray (Easy-spray ion source, Thermo Scientific) using a spray voltage = 1.7 kV and introduced into the mass spectrometer through a heated ion transfer tube (250 °C). Survey scans of peptide precursors in the *m/z* range 400–1600 were performed at resolution of 120,000 (@ 200 *m/z*) with a AGC target for Orbitrap survey of

2.0×10^5 and a maximum injection time of 50 ms. Tandem MS was performed by isolation at 1.6 Th with the quadrupole and high energy collisional dissociation (HCD) was performed in the Ion Routing Multipole (IRM), using a normalized collision energy of 35 and rapid scan MS analysis in the ion trap. The MS^2 ion count target was set to 10^2 and the maximum injection time was 250 ms. Only those precursors with charge state 1–3 and an intensity above the threshold of $5 \cdot 10^3$ were sampled for MS^2. The dynamic exclusion duration was set to 25 s with a 10 ppm tolerance around the selected precursor and its isotopes. Monoisotopic precursor selection was turned on. AGC target and maximum injection time (ms) for MS/MS spectra were 1.0×10^4 and 300, respectively. The instrument was run in top speed mode with 3 s cycles, meaning the instrument would continuously perform MS^2 events until the list of non-excluded precursors diminishes to zero or 3 s, whichever is shorter. MS/MS spectral quality was enhanced enabling the parallelizable time option (i.e. by using all parallelizable time during full scan detection for MS/MS precursor injection and detection). Mass spectrometer calibration was performed using the Pierce® LTQ Velos ESI Positive Ion Calibration Solution (Thermo Fisher Scientific). MS data acquisition was performed using the *Xcalibur* v. 3.0.63 software (Thermo Fisher Scientific).

Database search. LC–MS/MS data were processed using Proteome Discoverer v. 1.4.1.14 (Thermo Scientific). Data were searched against the SwissProt database (release Gen 2015, containing 547,357 entries) using the MASCOT algorithm (Matrix Science, London, UK, version 2.5.1). The search was performed against Homo sapiens or rattus sequences database (20,200 and 7,930 sequences, respectively). Full tryptic or chymotryptic peptides with a maximum of 3 missed cleavage sites were subjected to bioinformatic search. Cysteine carboxyamidomethylation was set as fixed modification, except when unreduced and unalkylated protein was analyzed (in-gel digestion, second procedure), whereas acetylation of protein N-terminal, oxidation, dioxidation and trioxidation of cysteine, oxidation and dioxidation of methionine, and transformation of N-terminal glutamine and N-terminal glutamic acid residue in the pyroglutamic acid form were included as variable modifications. The precursor mass tolerance threshold was 10 ppm and the max fragment mass error was set to 0.6 Da. Peptide spectral matches (PSM) were validated using Target Decoy PSM Validator node based on q-values at a 1% FDR.

Protein preparation and thermal denaturation experiments

Wavelength scans were recorded from 203 – 260 nm at a scan speed of 100 nm/min, a data integration time of 1 s and averaged over three accumulations. Data were corrected for buffer contribution and smoothened using Means-Movement method. Thermal denaturation measurements were carried out as reported earlier [S3]. Briefly, protein unfolding was monitored at 215 nm, between 4 °C – 95 °C, at a ramp rate of 1 °C/min. The ellipticity values were converted to unfolded fractions and fitted to a two-state equation [S3] to obtain the mid-point of thermal denaturation (*Tm*). Other thermal unfolding parameters, namely the temperature at which unfolding is nucleated (*Tm*-start) and the end temperature for protein unfolding (*Tm*-end), were calculated as reported [S4]. This is also illustrated in Supplem. Fig. 3. Protein concentrations for all CD measurements were maintained at 0.16 µg/µl in the refolding reaction containing 50 mM phosphate buffer pH 7.2, 2 mM DTT, 100 mM NaCl and 1% LDAO. Samples were analysed on SDS-PAGE before and after all measurements.

Molecular Dynamics simulations

The channel was embedded in a POPE bilayer and hydrated in the presence of a 0.5 M concentration of KCl. After 200 steps of conjugate gradient minimization, the system was gradually heated in the NVT ensemble across the temperature progression 10, 100, 200, and 300 K for 50 ps, 1 ns, 5 ns, 10 ns respectively. In all of these equilibration stages a time-step of 2.0 fs was used to avoid possible steric clashes. The system was then further equilibrated at 300 K for 400 ns using a 4.0 fs time step and rescaling hydrogen mass by a factor of 4. Using as input the coordinates of the last frame of this trajectory, but assigning different velocities sampled from the Maxwell- Boltzmann distribution, we then performed 5 independent production runs of 100 ns each in the NVT ensemble. The structural analysis was performed on the last configuration of the fifth run. The Solvent Accessible Surface Area, computed with the Shrake-Rupley algorithm [S5] was averaged on the five runs and converted to the radius of a circle with the same area. All simulations were performed with the Amber ff99SBildn [S6] force field using the ACEMD [S7] molecular dynamics program.

SUPPLEMENTARY REFERENCES

S1. De Pinto V, Prezioso G, Palmieri F. A simple and rapid method for the purification of the mitochondrial porin from mammalian tissues. Biochim. Biophys. Acta. 1987; 905: 499-502.

S2. Menzel VA, Cassará MC, Benz R, de Pinto V, Messina A, Cunsolo V, Saletti R, Hinsch KD, Hinsch E. Molecular and functional characterization of VDAC2 purified from mammal spermatozoa. Biosci. Rep. 2009; 29: 351-362.

S3. Maurya SR, Mahalakshmi R. Modulation of human mitochondrial voltage-dependent anion channel 2 (hVDAC-2) structural stability by cysteine-assisted barrel-lipid interactions. J. Biol. Chem. 2013; 288: 25584-25592.

S4. Chaturvedi D, Mahalakshmi R. Juxtamembrane tryptophans have distinct roles in defining the OmpX barrel-micelle boundary and facilitating protein-micelle association. FEBS Lett. 2014; 588: 4464-4471.

S5. Shrake A, Rupley JA. Environment and exposure to solvent of protein atoms. Lysozyme and insulin. J. Mol. Biol. 1973; 79: 351-371.

S6. Lindorff-Larsen K, Piana S, Palmo K, Maragakis P, Klepeis JL, Dror RO, Shaw DE. Improved side-chain torsion potentials for the Amber ff99SB protein force field. Proteins. 2010; 78: 1950-1958.

S7. Harvey M, Giupponi G, De Fabritiis, G. Accelerated molecular dynamics simulations in the microsecond timescale. J. Chem. Theory and Comput. 2009; 5: 1632-1639.

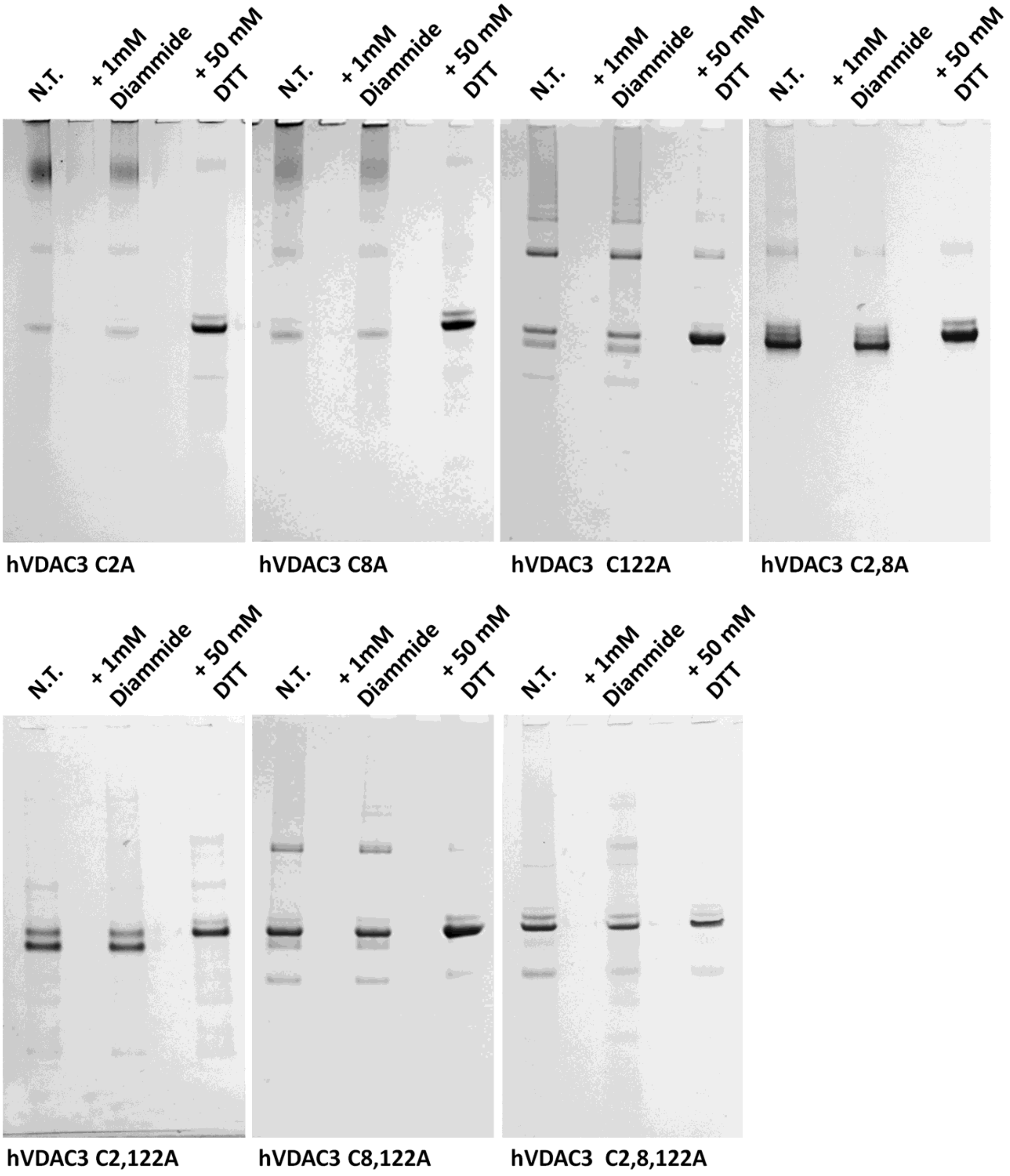

Supplementary Figure 1. Electrophoretic analysis of recombinant refolded hVDAC3 cysteine mutants produced in this work. Recombinant refolded hVDAC3 Cys-->Ala mutants C2A, C8A, C122A, C2,8A, C2,122A, C8,122A, and C2,8,122A were reduced with 50 mM DTT or oxidized with 1 mM diamide and run on a 12% Nu-Page Novex gel without any reducing agents.

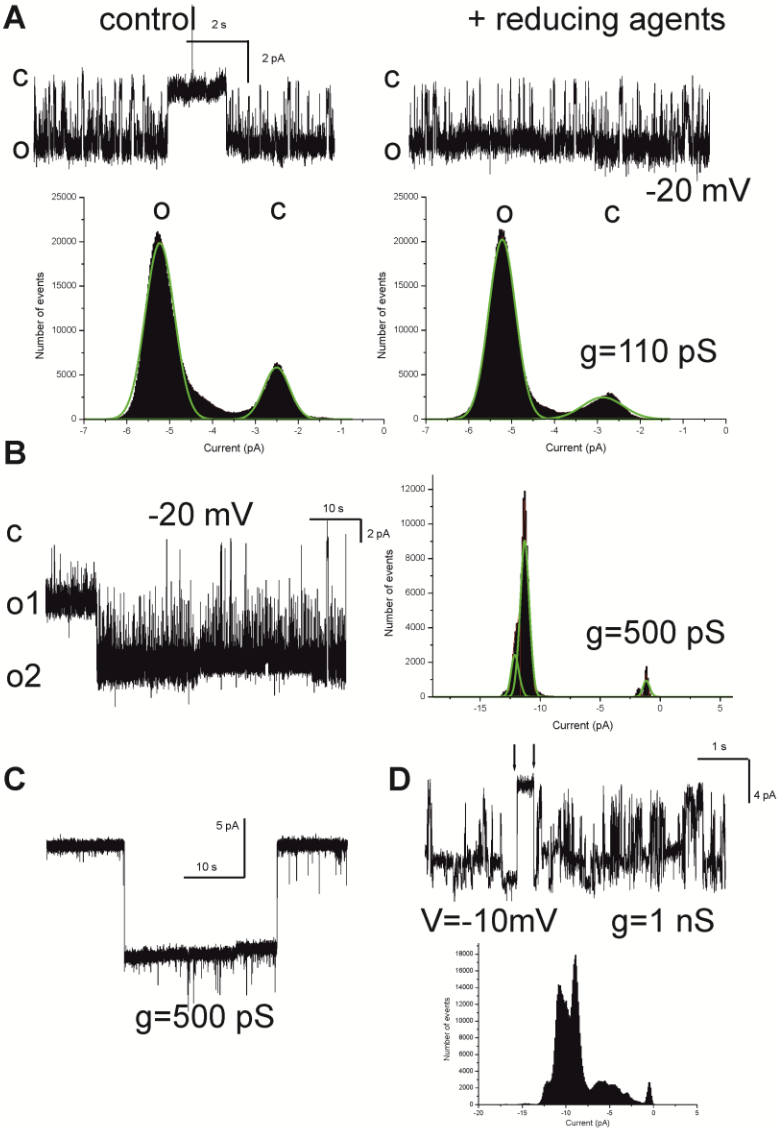

A
control
+ reducing agents
c
o
2 s
2 pA
-20 mV
o
c
g=110 pS
Number of events
Current (pA)
B
-20 mV
10 s
2 pA
c
o1
o2
g=500 pS
Number of events
Current (pA)
C
5 pA
10 s
g=500 pS
D
1 s
4 pA
V=-10mV
g=1 nS
Number of events
Current (pA)

E

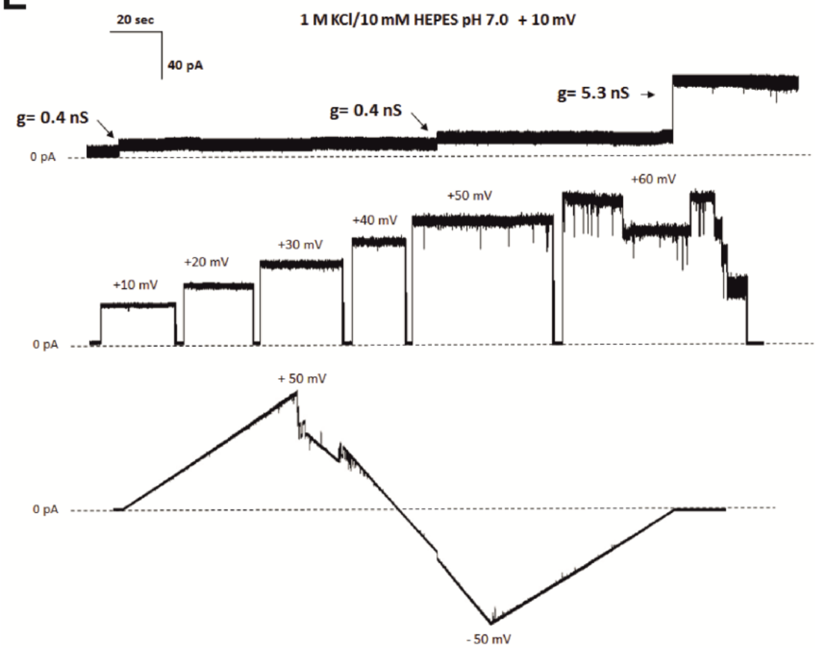

Supplementary Figure 2. Electrophysiological features of hVDAC3 in the presence of reducing agents. (A) Representative experiment showing current traces (upper parts) and respective amplitude histograms (lower parts) before (left panels) and after (right panel) addition of 10 mM DTT to both cis and trans chambers. Activity was measured in 1 M KCl, at pH 7.0. A slight increase in the relative intensity of the peaks for open (o) versus closed (c) state can be observed in the amplitude histograms obtained from 30 second-traces, indicating a slight increase in the open probability of the channel upon reduction. In 5 different experiments the open probability increased by 22±9 % upon addition of DTT to VDAC3. **(B)** Simultaneous gating of more open channels recorded upon 30 min. pre-incubation with 10 mM DTT and 10 mM β-mercaptoethanol gives an overall apparent conductance approaching 500 pS at -20 mV in the representative experiment shown (Vcis). Right part reports the amplitude histogram corresponding to the maximal conductance state (o2), while the left part illustrates channel activity revealing gating with lower, intermediate conductance (o1) as well. Single gating events of 500 pS as well as of 90 pS are resolvable in both the current trace and in the amplitude histogram. **(C)** A 500 pS step event with slow kinetics (typical of VDAC1) was observed only in 1 experiment performed as in (B) (Vcis= -20 mV). **(D)** Upper part: Current trace recorded under standard conditions showing multiple conductance states and gating events of 1 nS (arrows). Lower part: corresponding amplitude histograms illustrating that numerous distinct current levels co-exist throughout the experiment. **(E)** activity of VDAC3 purified and refolded in the presence of 1 mM DTT. Time scale is the same for the three current traces shown and is indicated in the upper part. Traces are representative of 3 experiments.

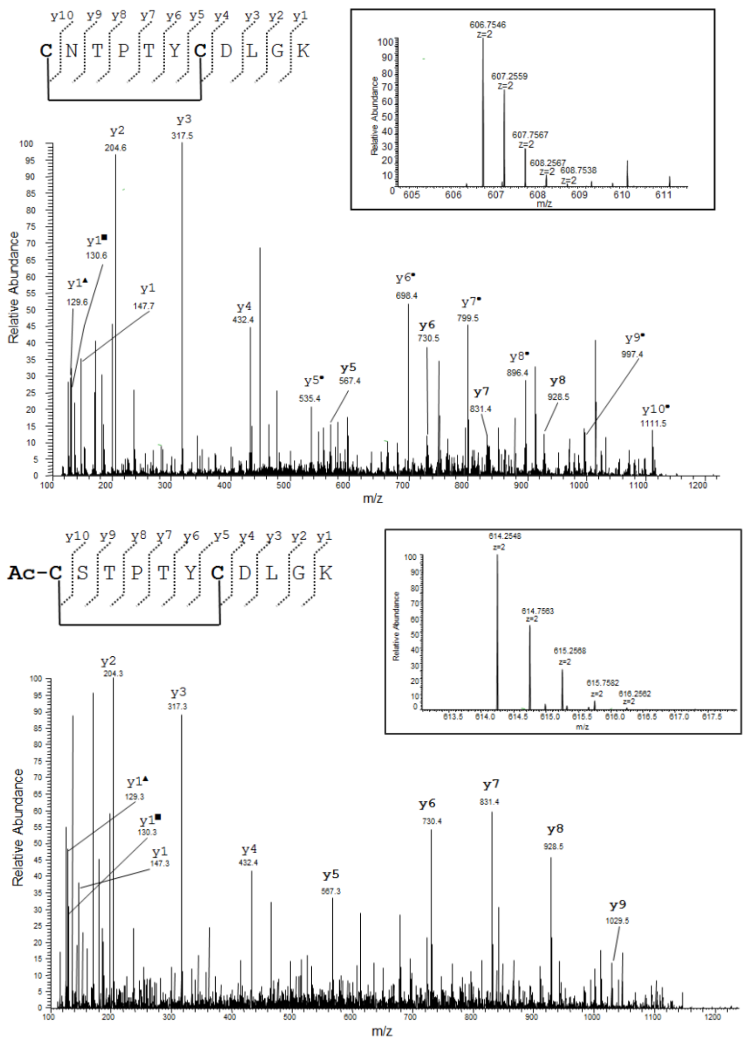

Supplementary Figure 3. MS/MS mass spectra showing the presence of a disulfide bond in the peptide 2-12 of the recombinant human and rat VDAC3. (A) MS/MS mass spectrum of the doubly charged molecular ion at m/z 606.7546 (calculated 606.7548) of the N-terminal non-acetylated tryptic peptide of recombinant hVDAC3 containing the disulfide bond between the cysteine residues 2 and 8. **(B)** MS/MS mass spectrum of the doubly charged molecular ion at m/z 614.2548 (calculated 614.2547) of the N-terminal acetylated tryptic peptide of rVDAC3 containing the disulfide bond between the cysteine residues 2 and 8. Fragment ions originated from the neutral loss of H_2O are indicated by ▲. Fragment ions originated from the neutral loss of NH_3 are indicated by ■. Fragment ions originated from the cleavage of -S-S- bond are indicated by ●. Fragment ions originated from the cleavage of $-CH_2$-S- bond are signed in bold. The full scan mass spectra of the molecular ions are shown in the insets.

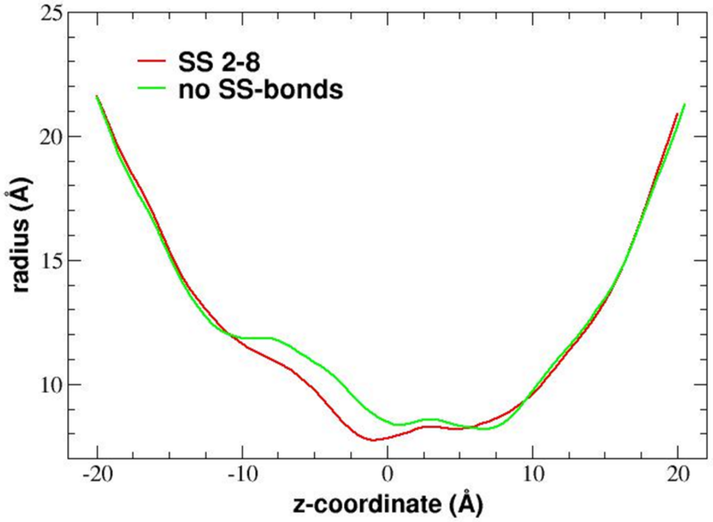

Supplementary Figure 4. Profiles of solvent accessible surface area along the axis of the VDAC3 channel.

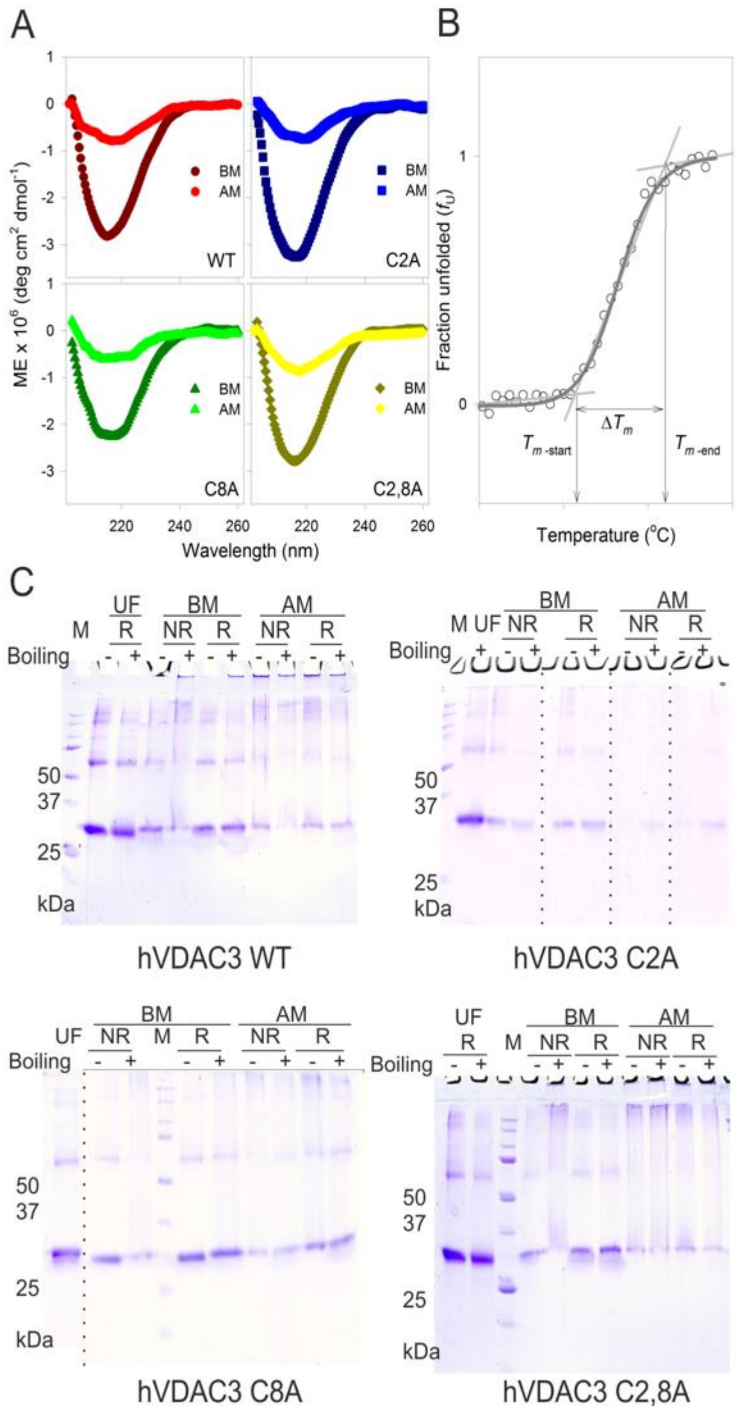

Supplementary Figure 5. Thermal denaturation studies of hVDAC3 and sample analysis. (A) Far-UV CD wavelength scans of WT (red circles), C2A (blue squares), C8A (green triangles) and C2,8A (olive diamonds) before (BM) and after (AM) thermal denaturation. Note that C8A shows marginally lower secondary structure content (BM profiles), when compared with the other proteins. All hVDAC3 protein constructs show a considerable loss in secondary structure content after thermal denaturation. ME: Molar ellipticity. (B) Schematic representation of a thermal denaturation profile (open circles) fitted to a two-state equation (solid black sigmoidal line) is used as an illustration to show how different parameters ($T_{m\text{-start}}$, $T_{m\text{-end}}$ and Δ

T_m) are derived from fits of the various data segments to a polynomial linear function (gray solid lines). Black drop lines with arrows show $T_{m\text{-start}}$, $T_{m\text{-end}}$ and ΔT_m calculated from the intercepts of the fits. (C) SDS-PAGE analysis of protein before and after thermal denaturation. Refolded samples were treated with non-reducing (NR) or reducing (R) dye and loaded on the gel without (-) or with boiling for 3 min at 100 °C (+). BM samples are largely monomeric whereas AM samples are largely aggregated and reside either in the wells or migrate as higher order bands. Images from different gels that are presented together are separated by a dotted line. M: molecular weight marker; important molecular weights are indicated; UF: urea-unfolded hVDAC3 control.
